# Comprehensive analysis of risk factors for intracranial aneurysm rupture: a retrospective cohort study

**DOI:** 10.3389/fneur.2025.1559484

**Published:** 2025-03-31

**Authors:** Bin Zhang, Zisheng Liu, Jiaming Xu, Jianyong Cai, Huajun Ba, Qun Lin, Jun Sun, Liangzhi Ye

**Affiliations:** Panvascular Disease Management Center, Wenzhou Central Hospital, Wenzhou, Zhejiang, China

**Keywords:** intracranial aneurysms, inflammatory markers, metabolic biomarkers, coagulation factors, predictive modeling

## Abstract

**Background:**

Intracranial aneurysms (IAs) can lead to subarachnoid hemorrhage, a life-threatening event associated with high morbidity and mortality. Identifying individuals at elevated risk is crucial for guiding timely interventions and improving patient outcomes.

**Methods:**

In this retrospective cohort study, 850 patients who received interventional or surgical treatment for IAs between January 2018 and January 2024 were included. Demographic data (e.g., age, sex), lifestyle factors, and comorbidities were recorded. Hematologic, biochemical, and coagulation parameters were measured to evaluate their potential association with IA rupture. A univariate logistic regression was first conducted, followed by a multivariate logistic regression with a backward stepwise approach to derive the final predictive model. The model’s performance was assessed using the area under the receiver operating characteristic curve (AUC), calibration curves, and decision curve analysis.

**Results:**

Younger age, female sex, higher neutrophil count, lower hematocrit, and elevated markers of inflammation and coagulation (including fibrinogen and D-dimer) emerged as key risk factors. Electrolyte imbalances, such as low potassium, and elevated lactate dehydrogenase were also significantly associated with rupture. The optimized model achieved an AUC of 0.815, with good calibration and clinical utility indicated by decision curve analysis.

**Conclusion:**

These findings highlight the interplay of demographic, inflammatory, metabolic, and coagulation parameters in determining rupture risk in patients with IAs. Incorporating these risk factors into clinical practice may enhance early detection, guide targeted prevention strategies, and ultimately improve outcomes for high-risk individuals.

## 1 Introduction

Intracranial aneurysms (IAs), defined as abnormal dilations of the cerebral arteries, represent a substantial public health concern because their rupture can precipitate subarachnoid hemorrhage (SAH), a severe condition marked by elevated morbidity and mortality rates (1). Affecting an estimated 1 to 6% of the general population, IAs carry an early mortality rate of 25 to 50% upon rupture, highlighting the considerable burden of this disease on patients and healthcare systems (2). The spontaneous rupture of an IA is considered a critical neurosurgical emergency, often resulting in severe neurological deficits, cognitive impairment, and, in many cases, fatality (3). Despite significant advancements in both endovascular and surgical treatments, managing ruptured aneurysms remains a complex clinical challenge due to the variability in aneurysm behavior and the diverse clinical presentations of affected patients (4). Large-scale clinical trials, such as the International Study of Unruptured Intracranial Aneurysms (ISUIA) and the International Subarachnoid Aneurysm Trial (ISAT), have provided essential insights into the factors related to aneurysm formation, growth, and rupture, yet there remains an ongoing need for a deeper understanding of these factors to better inform treatment strategies (5–7).

Several risk factors for IA rupture have been identified in prior studies, including demographic characteristics, lifestyle factors, and clinical conditions (8). Hypertension, smoking, and a family history of aneurysms have been consistently identified as key risk factors for rupture (8–10). These findings underscore the importance of understanding the heterogeneous nature of IA pathogenesis. However, the interplay between these risk factors and the variation in aneurysm characteristics highlights the need for individualized risk assessment frameworks. Furthermore, population-based cohort studies suggest that genetic and environmental factors may also significantly contribute to the rupture risk, emphasizing the importance of monitoring high-risk individuals and the potential value of tailored preventive strategies (10, 11).

The primary aim of this study is to evaluate the impact of a comprehensive range of factors—including demographic characteristics, lifestyle habits, clinical comorbidities, hematologic markers, biochemical parameters, and coagulation profiles—on the likelihood of IA rupture. By systematically analyzing these diverse influences, this study seeks to provide robust evidence to refine clinical decision-making and improve risk stratification. A clearer understanding of these determinants could aid clinicians in identifying high-risk patients and implementing more effective preventive strategies.

This study holds significant potential for enhancing early prevention and management of intracranial aneurysms. By elucidating the metabolic and coagulation markers linked to IA rupture, we aim to expand current risk stratification frameworks and support precision-guided preventive measures in populations at highest risk.

## 2 Methods

### 2.1 Study design and population

**Figure 1:**
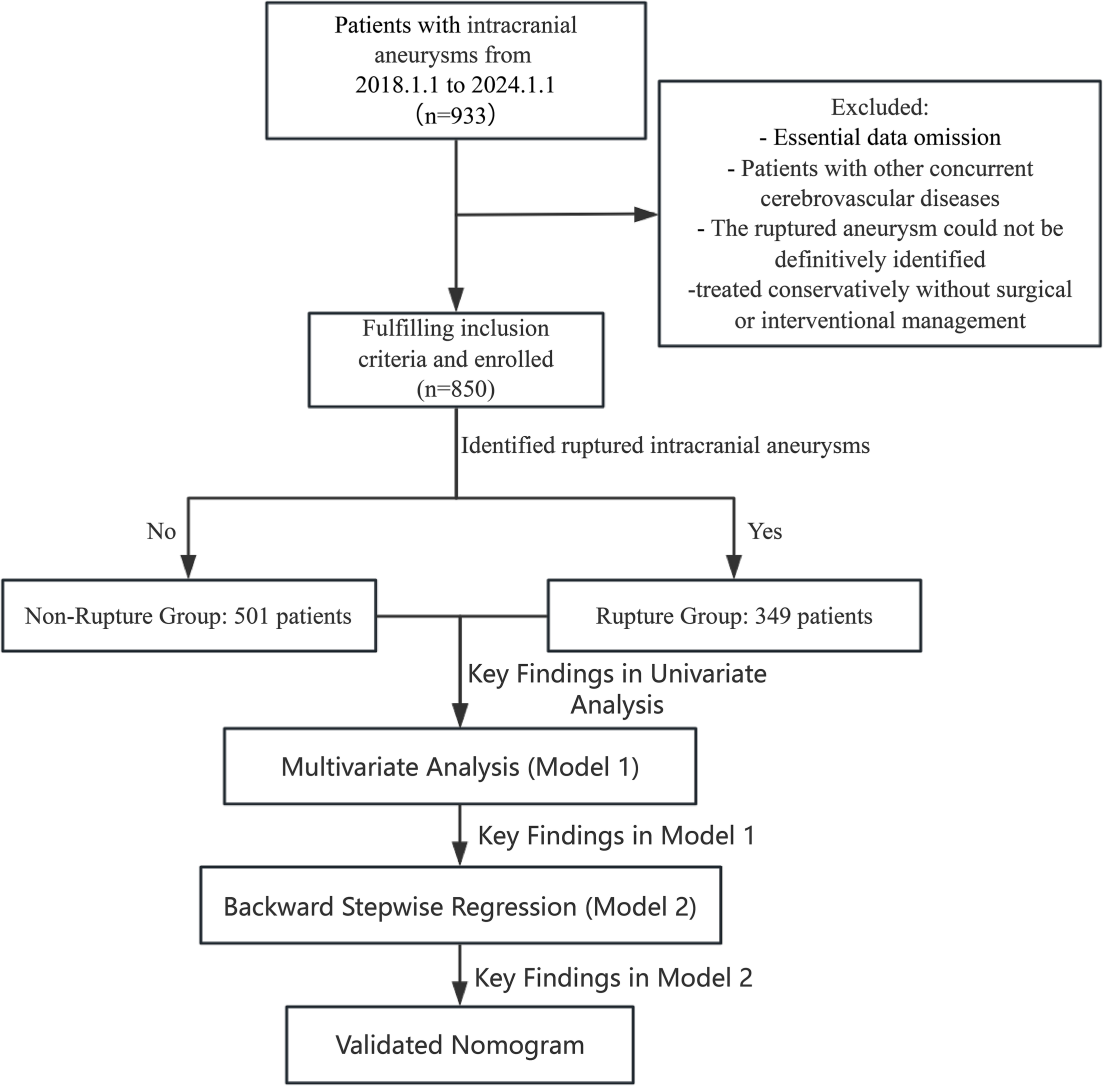
Flowchart depicting the patient selection process. This retrospective cohort study included 933 patients with intracranial aneurysms from January 1, 2018 to January 1, 2024. After applying inclusion and exclusion criteria, 850 patients were enrolled. Patients were stratified into two groups based on rupture status: Non-Ruptured Group (*n* = 501) and Ruptured Group (*n* = 349). The study’s analytical workflow, including univariate analysis, multivariate regression (Model 1), backward stepwise regression (Model 2), and nomogram validation is outlined.

This retrospective cohort study included patients who underwent intracranial aneurysm interventional treatment or surgical clipping at Wenzhou Central Hospital between January 2018 and January 2024. Patients were classified into two groups: those with ruptured intracranial aneurysms (rupture group) and those with unruptured intracranial aneurysms (non-rupture group). Aneurysm-specific characteristics—such as size, location, and morphology—are well-known predictors of rupture risk. However, because this was a retrospective study and detailed measurements and morphological data for aneurysms were not consistently documented, precise parameters (e.g., exact aneurysm size and specific morphological features) were unavailable for all included patients. But as shown in Table 1, our department employs a consensus-driven approach to determine surgical intervention for unruptured intracranial aneurysms, reached through comprehensive preoperative discussions involving the entire neurosurgical team. Consequently, we excluded patients who did not meet these indications of surgery. The unruptured aneurysm cohort therefore consisted only of patients who ultimately underwent surgical or endovascular intervention due to high-risk criteria (e.g., aneurysm ≥5 mm, posterior circulation, irregular morphology, or documented growth). Although this selection helps create a more comparable group to ruptured aneurysms, the absence of detailed aneurysm-specific data—along with the potential discrepancy between pre- and post-rupture findings—remains a key limitation of our analysis. Inclusion criteria were as follows: (1) Patients with a confirmed diagnosis of intracranial aneurysms based on imaging studies, including computed tomography angiography (CTA), magnetic resonance angiography (MRA), or digital subtraction angiography (DSA). (2) Patients who underwent definitive aneurysm treatment, either through endovascular techniques or surgical clipping. (3) Availability of complete demographic, clinical, laboratory, and imaging data. Exclusion criteria included: (1) Patients with incomplete or missing medical records or follow-up data. Patients with concurrent cerebrovascular diseases, such as arteriovenous malformations (AVMs) or Moyamoya disease, which could confound aneurysm-related outcomes. (2) The ruptured aneurysm could not be definitively identified. (3) Patients treated conservatively without surgical or interventional management. Aneurysms associated with infectious or inflammatory etiologies (e.g., mycotic aneurysms). The final dataset included only patients meeting these strict criteria to ensure robust and reliable analysis. This retrospective study adheres to the principles outlined in the Declaration of Helsinki and received ethical clearance from the institutional review board, which also granted a waiver for the requirement of informed consent due to the retrospective nature of the study (Figure 1).

**Table 1:** Departmental indications for endovascular intervention/surgery in unruptured intracranial aneurysms.

Parameter	Criteria	Departmental recommendation
Aneurysm size	<5 mm (anterior circulation)	Typically managed by observation due to relatively low rupture risk.
	5–7 mm	Consider endovascular intervention/surgery if additional risk factors (e.g., documented expansion) are present.
	≥7 mm	Endovascular intervention/surgery recommended, as the rupture risk substantially increases beyond this threshold.
	≥10 mm	Strongly recommended for endovascular intervention/surgery.
	≥25 mm (giant)	Urgent or proactive endovascular intervention/surgery due to a very high rupture risk.
Aneurysm location	Posterior circulation (e.g., vertebral artery, basilar artery)	Lower threshold for intervention owing to higher baseline rupture risk; may be indicated even if <5 mm.
	Basilar apex	Particularly prone to rupture; typically warrants endovascular intervention/surgery regardless of size.
	Anterior circulation	Usually observed if <5 mm; recommend endovascular intervention/surgery if ≥5 mm or if morphology is high risk.
Aneurysm morphology	Irregular shape (e.g., lobulated, saccular, intramural thrombus)	Suggests wall instability; generally indicates endovascular intervention/surgery.
	Wide-neck (neck width ≥ 50% of aneurysm diameter)	May require advanced endovascular techniques (e.g., stent-assisted or balloon-assisted coiling) or meticulous surgical clipping.
	Documented interval growth	Endovascular intervention/surgery recommended, even if initial size was small.

All decisions are made through a comprehensive departmental review, ensuring uniform application of these criteria.

These guidelines reflect individual institution’s practice patterns and may not represent universal standards.

### 2.2 Data collection

Patient data were extracted from electronic medical records and included a comprehensive array of demographic, lifestyle, clinical, hematologic, biochemical, and coagulation parameters. Demographic variables encompassed age, sex, birthdate (used to calculate age), height, weight, body mass index (BMI), and body surface area (BSA, calculated using the Dubois formula). Lifestyle factors, including smoking and alcohol consumption, were documented as categorical variables. Clinical data included comorbidities such as hypertension, diabetes mellitus, and coronary artery disease, given their established association with vascular pathology.

Hematologic data included systemic inflammatory markers and blood health indicators: white blood cell (WBC) count, red blood cell (RBC) count, hemoglobin (Hb), and platelet count (PLT). WBC subtypes—neutrophil, lymphocyte, monocyte, eosinophil, and basophil counts, as well as their respective percentages—were also evaluated. Red blood cell indices, such as mean corpuscular volume (MCV), mean corpuscular hemoglobin concentration (MCHC), mean corpuscular hemoglobin (MCH), and red cell distribution width (RDW), were recorded. Platelet parameters, including mean platelet volume (MPV), platelet distribution width (PDW), and plateletcrit (PCT), were analyzed for their potential role in rupture risk.

Biochemical markers assessed renal, metabolic, and systemic function. Parameters included creatinine, glucose, and electrolytes (sodium, potassium, chloride), as well as liver function tests—alanine aminotransferase (ALT) and total bilirubin (TBIL). Other biochemical markers, such as total protein (TP), lactate dehydrogenase (LDH), and calcium (Ca), were also included. Laboratory measurements were standardized and performed using calibrated equipment to ensure accuracy.

To evaluate the coagulation profile, key parameters such as prothrombin time (PT), international normalized ratio (PT-INR), activated partial thromboplastin time (APTT), thrombin time (TT), fibrinogen (Fbg), and D-dimer levels were assessed. Additional coagulation-related ratios, including thrombin time ratio (TTR) and activated partial thromboplastin time ratio (APTTR), were analyzed to identify potential hypercoagulability or coagulopathy that could contribute to aneurysm rupture risk.

### 2.3 Statistical analysis

Descriptive statistics were used to summarize demographic, clinical, laboratory, and coagulation data. Continuous variables were reported as means ± standard deviations, while categorical variables were expressed as frequencies and percentages. Comparisons between the rupture and non-rupture groups were conducted using t-tests for continuous variables and chi-square tests for categorical variables. Univariate logistic regression analysis was performed to assess the association of each variable with aneurysm rupture risk. Variables with a *p*-value <0.1 in the univariate analysis were included in the multivariate logistic regression model, with a backward stepwise regression approach used to identify independent predictors of rupture. The backward stepwise approach prioritized variables based on their clinical relevance and statistical significance, ensuring that only robust predictors were retained in the final model. Odds ratios (ORs) with 95% confidence intervals (CIs) were calculated.

To visualize the predictive model, a nomogram was developed based on significant predictors from the multivariate analysis. Model performance was evaluated using the receiver operating characteristic (ROC) curve, with the area under the curve (AUC) indicating predictive accuracy. Calibration was assessed using calibration plots, and clinical utility was examined through decision curve analysis (DCA).

All statistical analyses were conducted using the Statistical Package for the Social Sciences (SPSS) version 27.0, including the backward stepwise regression model, and R software (versions 1.6 and 4.1.3; Foundation for Statistical Computing, Vienna, Austria), with a significance level set at *p* < 0.05.

## 3 Results

### 3.1 Baseline

A total of 850 patients were included in this study, comprising 439 males (51.65%) and 411 females (48.35%). The mean age of the cohort was 59.31 ± 12.56 years, with an average height of 162.98 ± 9.19 cm and weight of 63.47 ± 10.54 kg. Among these patients, 466 (54.82%) had a history of hypertension, 150 (17.65%) had diabetes mellitus, and 41 (4.82%) had coronary artery disease. Additionally, 176 patients (20.71%) reported a history of smoking, and 116 (13.65%) reported alcohol consumption. Comparison of the rupture group (*n* = 349) and the non-rupture group (*n* = 501) revealed significant gender and age differences. A higher proportion of females was observed in the rupture group compared to the non-rupture group (54.15% vs. 44.31%, *p* = 0.006). Patients in the rupture group were significantly younger than those in the non-rupture group (56.21 ± 13.36 years vs. 61.47 ± 11.51 years, *p* < 0.001). Hypertension was less prevalent in the rupture group compared to the non-rupture group (48.71% vs. 59.08%, *p* = 0.004). However, no significant differences were observed between the groups regarding BMI, body surface area, smoking status, or alcohol consumption (Table 2).

**Table 2:** Baseline of demographic and clinical characteristics.

Variable	Total (*n* = 850)	Non-ruptured (*n* = 501)	Ruptured (*n* = 349)	*p*-value
Gender				0.006
Male	439 (51.65%)	279 (55.69%)	160 (45.85%)	
Female	411 (48.35%)	222 (44.31%)	189 (54.15%)	
Age (years)	59.31 ± 12.56	61.47 ± 11.51	56.21 ± 13.36	<0.001
Height (cm)	162.98 ± 9.19	162.80 ± 10.23	163.23 ± 7.44	0.479
Weight (kg)	63.47 ± 10.54	63.32 ± 10.71	63.70 ± 10.29	0.598
BMI	26.83 ± 88.48	28.89 ± 115.21	23.86 ± 3.19	0.33
BSA (m^2^)	1.68 ± 0.16	1.68 ± 0.17	1.68 ± 0.15	0.466
Smoking history				0.132
No	674 (79.29%)	388 (77.45%)	286 (81.95%)	
Yes	176 (20.71%)	113 (22.55%)	63 (18.05%)	
Alcohol consumption				0.859
No	734 (86.35%)	434 (86.63%)	300 (85.96%)	
Yes	116 (13.65%)	67 (13.37%)	49 (14.04%)	
Hypertension				0.004
No	384 (45.18%)	205 (40.92%)	179 (51.29%)	
Yes	466 (54.82%)	296 (59.08%)	170 (48.71%)	
Diabetes mellitus				0.065
No	700 (82.35%)	402 (80.24%)	298 (85.39%)	
Yes	150 (17.65%)	99 (19.76%)	51 (14.61%)	
CAD				0.588
No	809 (95.18%)	479 (95.61%)	330 (94.56%)	
Yes	41 (4.82%)	22 (4.39%)	19 (5.44%)	

BMI, body mass index; BSA. body surface area; CAD, coronary artery disease.

Significant differences were also observed in laboratory and biochemical parameters. The rupture group demonstrated higher white blood cell counts (10.31 ± 4.55 vs. 7.37 ± 2.85, *p* < 0.001) and neutrophil counts (7.85 ± 4.47 vs. 4.79 ± 2.61, *p* < 0.001) compared to the non-rupture group. Conversely, red cell distribution width (RDW) was lower in the rupture group (13.08 ± 1.30 vs. 12.90 ± 0.93, *p* = 0.023), while platelet distribution width (PDW) was higher (14.34 ± 2.47 vs. 13.36 ± 2.78, *p* < 0.001). Among the biochemical markers, glucose (7.74 ± 3.13 vs. 6.81 ± 2.82, *p* < 0.001), lactate dehydrogenase (205.97 ± 60.65 vs. 186.87 ± 54.57, *p* < 0.001), and D-dimer levels (median 336.00 ng/mL vs. 172.00 ng/mL, *p* < 0.001) were significantly elevated in the rupture group (Table 3).

**Table 3:** Baseline of laboratory and biochemical characteristics.

Variable	Ruptured (*n* = 349)	Non-ruptured (*n* = 501)	*p*
WBC count (10^9/L)	10.31 ± 4.55	7.37 ± 2.85	<0.001
RBC count (10^12/L)	4.44 ± 0.52	4.49 ± 0.58	0.169
MCV (fL)	90.12 ± 5.84	91.13 ± 5.64	0.012
MCHC (g/L)	334.07 ± 11.95	334.40 ± 11.41	0.691
MCH (pg)	30.14 ± 2.46	30.50 ± 2.24	0.032
RDW (%)	13.08 ± 1.30	12.90 ± 0.93	0.023
Lymphocyte count (10^9/L)	1.82 ± 1.19	1.92 ± 0.81	0.186
Monocyte count (10^9/L)	0.53 ± 0.25	0.50 ± 0.21	0.061
Neutrophil count (10^9/L)	7.85 ± 4.47	4.79 ± 2.61	<0.001
Hematocrit (%)	39.96 ± 4.78	40.81 ± 4.81	0.011
Eosinophil percentage (%)	1.10 ± 1.49	2.12 ± 1.89	<0.001
Basophil percentage (%)	0.28 ± 0.26	0.39 ± 0.27	<0.001
Hemoglobin (g/L)	133.66 ± 17.87	136.50 ± 17.00	0.02
Platelet count (10^9/L)	225.88 ± 62.36	226.43 ± 63.54	0.9
MPV (fL)	9.89 ± 1.24	10.16 ± 1.21	0.002
PDW (%)	14.34 ± 2.47	13.36 ± 2.78	<0.001
Plateletcrit (%)	0.22 ± 0.06	0.23 ± 0.06	0.231
ALT (U/L)	26.98 ± 21.60	28.23 ± 46.06	0.595
Total bilirubin (μmol/L)	11.79 ± 5.75	12.92 ± 8.05	0.017
Total protein (g/L)	73.09 ± 8.23	71.38 ± 6.97	0.002
Creatinine (μmol/L)	78.99 ± 72.79	65.80 ± 27.75	<0.001
Potassium (mmol/L)	3.65 ± 0.53	3.86 ± 0.42	<0.001
Chloride (mmol/L)	103.77 ± 3.59	104.48 ± 2.77	0.002
Sodium (mmol/L)	139.36 ± 3.32	140.04 ± 2.70	0.002
Total calcium (mmol/L)	2.25 ± 0.13	2.28 ± 0.10	<0.001
LDH (U/L)	205.97 ± 60.65	186.87 ± 54.57	<0.001
Glucose (mmol/L)	7.74 ± 3.13	6.81 ± 2.82	<0.001
PT-INR	1.02 ± 0.15	1.02 ± 0.18	0.956
PT (s)	11.25 ± 1.73	11.24 ± 1.98	0.899
PT activity (%)	106.88 ± 14.29	107.92 ± 16.01	0.319
TT (s)	15.89 ± 3.80	16.38 ± 6.30	0.162
TT ratio	1.06 ± 0.22	1.06 ± 0.41	0.851
APTT (s)	29.78 ± 10.51	30.85 ± 4.60	0.073
Fibrinogen (g/L)	3.16 ± 0.84	3.31 ± 0.84	0.007
D-dimer (mg/L)	336 (167, 758)	172 (92, 314)	<0.001
WBC count (10^9/L)	10.31 ± 4.55	7.37 ± 2.85	<0.001
RBC count (10^12/L)	4.44 ± 0.52	4.49 ± 0.58	0.169
MCV (fL)	90.12 ± 5.84	91.13 ± 5.64	0.012
MCHC (g/L)	334.07 ± 11.95	334.40 ± 11.41	0.691
MCH (pg)	30.14 ± 2.46	30.50 ± 2.24	0.032
RDW (%)	13.08 ± 1.30	12.90 ± 0.93	0.023

WBC, white blood cell; RBC, red blood cell; MCV, mean corpuscular volume; MCHC, mean corpuscular hemoglobin concentration; MCH, mean corpuscular hemoglobin; RDW, red cell distribution width; MPV, mean platelet volume; PDW, platelet distribution width; ALT, alanine aminotransferase; LDH, lactate dehydrogenase; PT-INR, prothrombin time-international normalized ratio; PT, prothrombin time; TT, thrombin time; APTT, activated partial thromboplastin time.

### 3.2 Univariate logistic regression analysis

Univariate logistic regression analysis identified several factors associated with aneurysm rupture risk. Female gender was associated with a higher risk of rupture (OR: 1.48, 95% CI: 1.13–1.95, *p* = 0.005), and age was inversely associated with rupture risk (OR: 0.97, 95% CI: 0.96–0.98, *p* < 0.001). Hypertension (OR: 0.66, 95% CI: 0.50–0.87, *p* = 0.003), white blood cell count (OR: 1.26, 95% CI: 1.21–1.32, *p* < 0.001), and glucose levels (OR: 1.11, 95% CI: 1.06–1.17, *p* < 0.001) were also significant predictors. Other significant markers included neutrophil count, RDW, PDW, lactate dehydrogenase, and D-dimer (Table 4).

**Table 4:** Analysis for aneurysm rupture risk.

Variable	Unadjusted		Adjusted to Model 1		Adjusted to Model 2	
	OR (95% CI)	*p*_value	OR (95% CI)	*p*_value	OR (95% CI)	*p*_value
Female vs. Male	1.48 [1.13; 1.95]	0.005	0.93 (0.61, 1.43)	0.754		
Age	0.97 [0.96; 0.98]	<0.001	0.97 (0.95, 0.98)	<0.001	0.97 (0.95, 0.98)	<0.001
Height	1.01 [0.99; 1.02]	0.504				
Weight	1.00 [0.99; 1.02]	0.6				
BMI	1.00 [0.99; 1.01]	0.662				
BSA	1.36 [0.59; 3.13]	0.474				
Smoking (yes vs. no)	0.76 [0.53; 1.07]	0.111				
Alcohol (yes vs. no)	1.06 [0.71; 1.57]	0.779				
Hypertension	0.66 [0.50; 0.87]	0.003	1.18 (0.83, 1.69)	0.365		
Diabetes mellitus	0.70 [0.48; 1.00]	0.052				
CAD	1.25 [0.66; 2.36]	0.484				
WBC count	1.26 [1.21; 1.32]	<0.001	0.88 (0.74, 1.05)	0.155		
RBC count	0.84 [0.66; 1.08]	0.177				
MCV	0.97 [0.95; 0.99]	0.012	1.00 (0.78, 1.29)	0.979		
MCHC	1.00 [0.99; 1.01]	0.688				
MCH	0.94 [0.88; 0.99]	0.031	0.98 (0.47, 2.06)	0.966		
RDW	1.16 [1.03; 1.32]	0.018	1.17 (0.97, 1.43)	0.108		
NC	1.30 [1.24; 1.37]	<0.001	1.40 (1.16, 1.70)	0.001	1.30 (1.23, 1.38)	<0.001
Hct	0.96 [0.94; 0.99]	0.012	0.94 (0.54, 1.64)	0.834	0.94 (0.90, 0.94)	<0.001
Eos%	0.64 [0.58; 0.72]	<0.001	0.92 (0.81, 1.05)	0.205		
Baso%	0.16 [0.09; 0.30]	<0.001	0.89 (0.44, 1.81)	0.743		
Hb	0.99 [0.98; 1.00]	0.02	1.01 (0.85, 1.19)	0.952		
PLT count	1.00 [1.00; 1.00]	0.9				
MPV	0.84 [0.75; 0.94]	0.002	0.96 (0.83, 1.12)	0.61		
PDW	1.15 [1.09; 1.21]	<0.001	1.02 (0.95, 1.10)	0.543		
PCT	0.25 [0.02; 2.51]	0.236				
ALT	1.00 [1.00; 1.00]	0.641				
TBIL	0.97 [0.95; 1.00]	0.028	0.98 (0.96, 1.01)	0.119		
TP	1.03 [1.01; 1.05]	0.001	1.01 (0.98, 1.04)	0.698		
Cr	1.01 [0.99; 1.02]	<0.001	1.01 (0.99; 1.02)	0.001	1.01 (0.99; 1.02)	<0.001
K	0.37 [0.27; 0.52]	<0.001	0.67 (0.44, 1.03)	0.068	0.63 (0.42, 0.92)	0.018
Cl	0.93 [0.89; 0.97]	0.001	0.95 (0.88, 1.02)	0.169		
Na	0.93 [0.88; 0.97]	0.001	1.00 (0.93, 1.08)	0.974		
Ca	0.10 [0.03; 0.34]	<0.001	0.27 (0.05, 1.56)	0.143		
LDH	1.01 [1.00; 1.01]	<0.001	1.00 (1.00, 1.01)	0.038	1.00 (1.00, 1.01)	0.027
Glu	1.11 [1.06; 1.17]	<0.001	1.02 (0.96, 1.09)	0.48		
PT-INR	1.02 [0.45; 2.31]	0.957				
PT	1.00 [0.93; 1.08]	0.901				
PTA	1.00 [0.99; 1.00]	0.329				
TT	0.98 [0.94; 1.01]	0.219				
TTR	0.97 [0.64; 1.45]	0.865				
APTT	0.96 [0.93; 1.00]	0.036	0.97 (0.95, 0.99)	0.007	0.97 (0.95, 0.99)	0.003
Fbg	0.79 [0.67; 0.94]	0.088	0.71 (0.57, 0.89)	0.003	0.74 (0.59, 0.91)	0.005
D-dimer	1.00 [1.00; 1.00]	<0.001	1.00 (1.00, 1.00)	<0.001	1.00 (1.00, 1.00)	<0.001

OR, Odds Ratio; BMI, body mass index; BSA, body surface area; CAD, Coronary Artery Disease; WBC, white blood cell count; RBC, red blood cell count; MCV, mean corpuscular volume; MCHC, mean corpuscular hemoglobin concentration; MCH, mean corpuscular hemoglobin; RDW, red cell distribution width; NC, neutrophil count; Hct, hematocrit; Eos%, eosinophil percentage; Baso%, basophil percentage; Hb, hemoglobin; PLT, platelet count; MPV, mean platelet volume; PDW, platelet distribution width; PCT, plateletcrit; ALT, alanine aminotransferase; TBIL, total bilirubin; TP, total protein; Cr, creatinine; K, potassium; Cl, chloride; Na, sodium; Ca, total calcium; LDH, lactate dehydrogenase; Glu, glucose; PT-INR, prothrombin time international normalized ratio; PT, prothrombin time; PTA, prothrombin time activity; TT, thrombin time; TTR, thrombin time ratio; APTT, activated partial thromboplastin time; Fbg, fibrinogen; D-dimer, D-dimer.

### 3.3 Multivariate logistic regression analysis (Model 1)

In the initial multivariate logistic regression model (Model 1), several factors were identified as significant predictors of aneurysm rupture with *p*-values less than 0.05. Younger age was associated with a reduced risk of rupture (OR: 0.97, 95% CI: 0.95–0.98, *p* < 0.001). Higher neutrophil count was a strong predictor of rupture risk (OR: 1.40, 95% CI: 1.16–1.70, *p* = 0.001). Additionally, lower hematocrit was associated with increased rupture risk (OR: 0.94, 95% CI: 0.90–0.94, *p* < 0.001). Among biochemical markers, lactate dehydrogenase (OR: 1.00, 95% CI: 1.00–1.01, *p* = 0.038), D-dimer (OR: 1.00, 95% CI: 1.00–1.00, *p* < 0.001), and glucose levels (OR: 1.02, 95% CI: 1.00–1.04, *p* = 0.048) were also significantly associated with rupture risk. Potassium (OR: 0.67, 95% CI: 0.44–1.03, *p* = 0.068), fibrinogen (OR: 0.71, 95% CI: 0.57–0.89, *p* = 0.003), and APTT (OR: 0.97, 95% CI: 0.95–0.99, *p* = 0.007) were identified as additional significant predictors in this model. The likelihood ratio chi-square test for Model 1 was 309.42, with a *p*-value of <0.001, indicating a good model fit (Table 4).

### 3.4 Optimized model using backward stepwise regression (Model 2)

To refine the predictive model, a backward stepwise regression approach was employed, resulting in an optimized model (Model 2). In this model, significant predictors of aneurysm rupture included younger age (OR: 0.97, 95% CI: 0.95–0.98, *p* < 0.001), higher neutrophil count (OR: 1.30, 95% CI: 1.23–1.38, *p* < 0.001), lower hematocrit (OR: 0.94, 95% CI: 0.90–0.94, *p* < 0.001), and higher D-dimer levels (OR: 1.00, 95% CI: 1.00–1.00, *p* < 0.001). Potassium (OR: 0.63, 95% CI: 0.42–0.92, *p* = 0.018), fibrinogen (OR: 0.74, 95% CI: 0.59–0.91, *p* = 0.005), and APTT (OR: 0.97, 95% CI: 0.95–0.99, *p* = 0.003) also remained significant predictors in the optimized model. The likelihood ratio chi-square test for Model 2 was 288.154, with a *p*-value of <0.001, further confirming the good model fit after optimization (Table 4).

### 3.5 Nomogram and model validation

**Figure 2:**
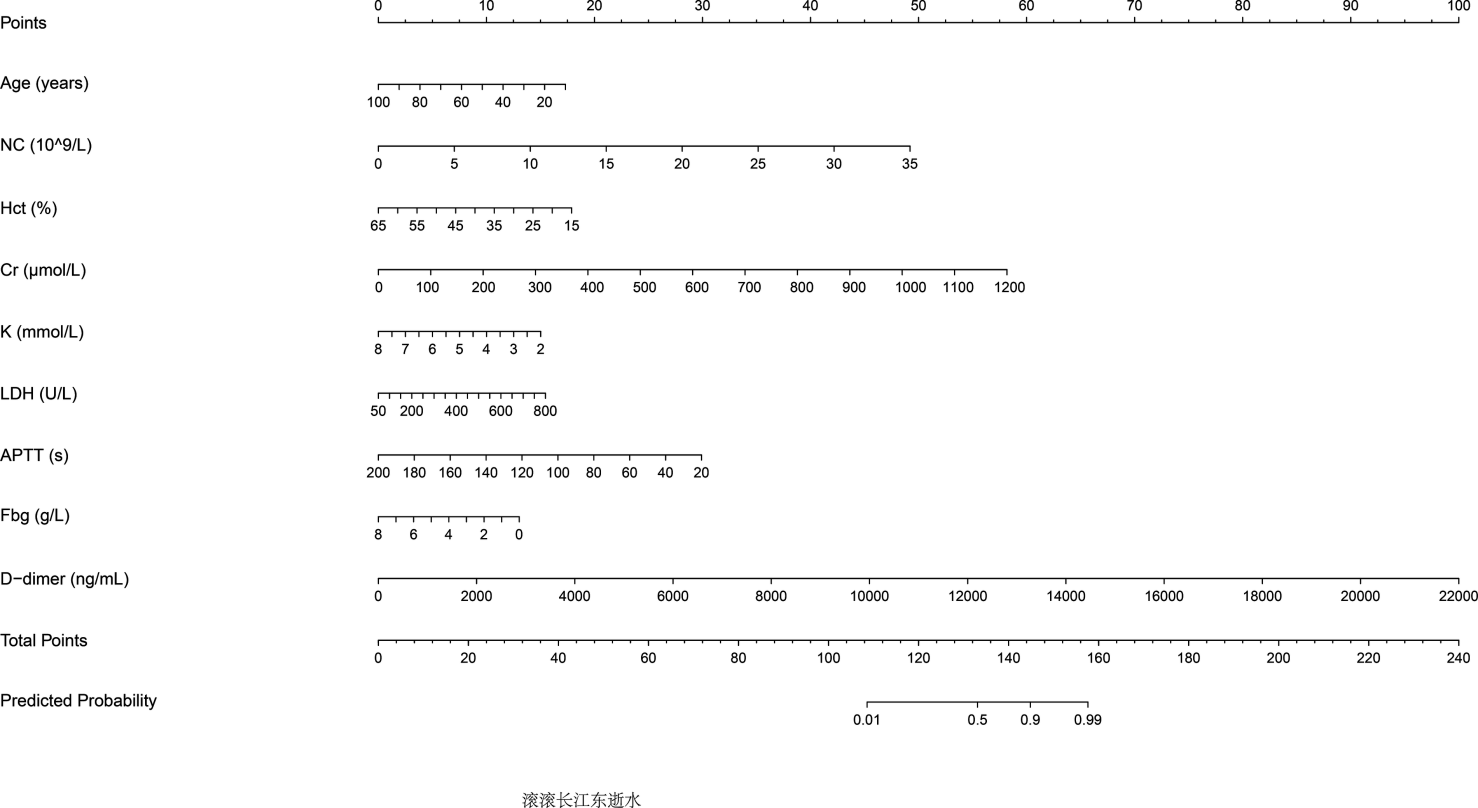
Nomogram for predicting aneurysm rupture risk. The nomogram developed based on Model 2 predictors is shown. Significant predictors include age, neutrophil count, hematocrit, fibrinogen, potassium, D-dimer levels, and activated partial thromboplastin time (APTT). The nomogram assigns scores to each predictor, which are summed to calculate the probability of aneurysm rupture.

**Figure 3:**
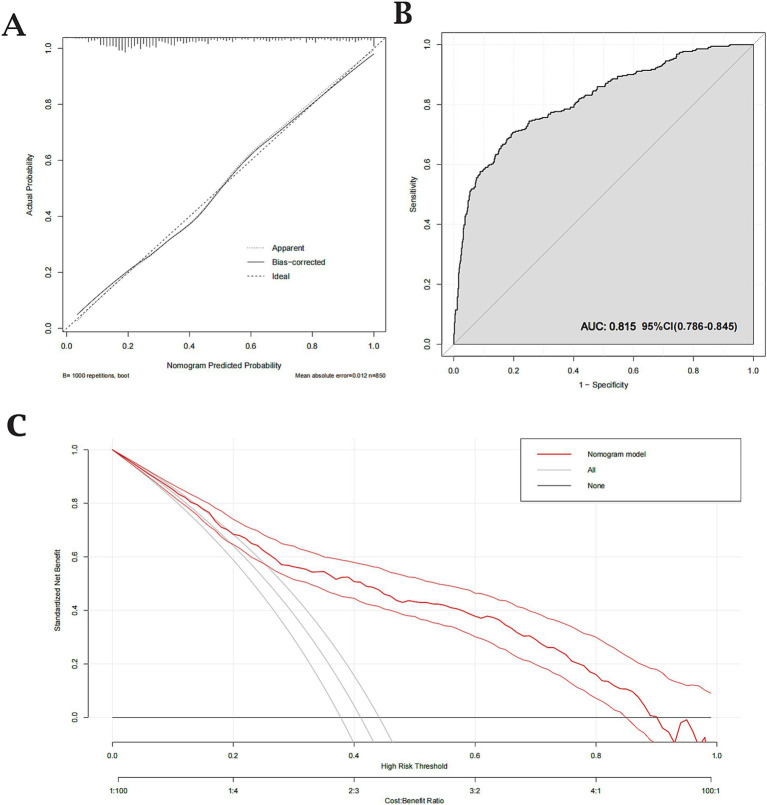
Calibration, Receiver Operating Characteristic (ROC), and Decision Curve Analysis (DCA) for the validated nomogram. **(A)** Calibration curve showing close agreement between predicted and observed probabilities (mean absolute error = 0.012). **(B)** ROC curve with an Area Under the Curve (AUC) of 0.815, indicating good discriminative ability (sensitivity = 0.802; specificity = 0.708). **(C)** DCA demonstrating the clinical utility of the nomogram across various threshold probabilities.

The predictive performance of Model 2 was visualized using a nomogram (Figure 2), which effectively illustrates the contribution of each significant predictor to the risk of aneurysm rupture. To further evaluate the model’s clinical utility, a calibration curve (Figure 3A) was used, demonstrating close alignment between the predicted probabilities and actual outcomes, with a mean absolute error of 0.012, indicating strong calibration accuracy. The ROC analysis (Figure 3B) yielded an AUC of 0.815 (95% CI: 0.786–0.845), with a sensitivity of 0.802 and a specificity of 0.708, demonstrating good discriminatory ability of the model. Finally, a DCA (Figure 3C) was conducted, showing positive net benefits across a range of threshold probabilities, indicating that Model 2 provides valuable decision-making support for predicting aneurysm rupture.

## 4 Discussion

This study identifies several independent risk factors associated with the rupture of intracranial aneurysms, including younger age, female gender, elevated inflammatory markers, and altered metabolic and coagulation profiles. Although several outcomes corroborate earlier reports, our data also reveal less-explored associations particularly involving potassium, creatinine, and lactate dehydrogenase (LDH) that warrant further investigation for their potential role in aneurysm rupture risk.

### 4.1 Age, gender-related factors and hypertension-related factors

Consistent with prior findings, our analysis indicates that younger age correlates with heightened IA rupture risk. In support of this observation, a meta-analysis by Vlak et al. reported an inverse correlation between age and rupture risk, implying that age-related vascular remodeling may mitigate rupture susceptibility (12). Further cohort research suggests that age-related vascular wall remodeling, associated with increased arterial stiffness, might buffer against abrupt pressure surges that otherwise precipitate rupture (13, 14).

Our results also indicate that women face a higher risk of aneurysm rupture, echoing earlier studies that link postmenopausal hormonal shifts to elevated rupture susceptibility in females. A study by Korja et al. observed that hormonal factors, including declining estrogen levels, may weaken the cerebral vasculature in women, predisposing them to rupture (15). Additional evidence indicates that sex-based variations in arterial wall thickness and elasticity may also contribute to the higher rupture propensity observed in females (16, 17).

Interestingly, our results showed an inverse relationship between a history of hypertension and rupture risk, a finding that contrasts with much of the existing literature. This discrepancy may be attributed to multiple factors. Long-term hypertension may lead to adaptive thickening of the vascular wall, a compensatory mechanism that enhances the vessel’s ability to withstand elevated pressures and reduces its susceptibility to rupture. Additionally, many patients with a history of hypertension in our study were likely receiving antihypertensive treatment, such as ACE inhibitors or calcium channel blockers, which are known to stabilize vascular walls by reducing inflammatory responses and oxidative stress (18, 19). These medications, combined with improved blood pressure control, may mitigate vascular stress and endothelial dysfunction, thereby lowering the risk of rupture (19). Although less frequently discussed, these protective mechanisms align with findings from studies on chronic hypertension and vascular remodeling, warranting further investigation to guide therapeutic strategies in high-risk populations (17, 20). This finding highlights the importance of optimizing antihypertensive therapies not only to manage systemic hypertension but also to potentially reduce the risk of aneurysm rupture.

### 4.2 Inflammatory, metabolic and biochemical factors

Elevated white blood cell and neutrophil counts underscore how systemic inflammation may destabilize aneurysm walls, aligning with existing evidence that links these immune cells to extracellular matrix degradation (21–23). Notably, neutrophil-driven inflammatory responses have been shown to degrade the extracellular matrix in vessel walls, thus heightening the potential for aneurysm rupture (21–23). We also observed that lower hematocrit levels may exacerbate endothelial hypoxia, weakening the vessel wall and thereby increasing rupture risk. While not extensively studied in intracranial aneurysms, similar links between low hematocrit and endothelial dysfunction are well-documented in peripheral vascular disorders (24).

Creatinine, a measure of renal function, also correlated with higher rupture risk, possibly reflecting how renal dysfunction amplifies endothelial damage and oxidative stress (19, 25, 26). Although direct evidence for creatinine’s role in IA rupture remains limited, studies in chronic kidney disease point to impaired vascular integrity under conditions of reduced renal function (27–29). Furthermore, lower potassium levels in the rupture group highlight the importance of electrolyte balance for maintaining stable vascular tone. Hypokalemia can amplify vascular smooth muscle contraction and arterial stiffness, potentially intensifying the stress on weakened vessel walls (30). Although data on potassium levels in IAs are sparse, research in hypertension and cardiovascular disease underscores that low potassium commonly correlates with increased vascular stiffness (31, 32).

LDH, a marker of cellular damage, was also elevated in the rupture group, suggesting a higher level of metabolic stress. Elevated LDH levels indicate tissue damage, which may reflect compromised vascular integrity in aneurysm walls (33). This marker is commonly elevated in ischemic events, where tissue hypoxia and cell damage are prominent (34). Its association with rupture risk here may imply similar underlying damage within aneurysm walls prior to rupture.

### 4.3 Coagulation factors

Alterations in coagulation, including elevated D-dimer and fibrinogen levels, were also noted. High fibrinogen levels are associated with increased blood viscosity and vascular stress, which can contribute to aneurysm instability. Elevated D-dimer, a fibrin degradation product, suggests a hypercoagulable state and has been associated with vascular damage in other cerebrovascular diseases (28). A shortened APTT further supports a pro-coagulant state, potentially destabilizing the aneurysm by promoting microthrombosis and local inflammatory responses (6).

### 4.4 Model validation

The final predictive model, validated by an AUC of 0.815 and supported by a well-calibrated nomogram and decision curve analysis, demonstrates strong predictive power and clinical applicability for assessing rupture risk.

### 4.5 Clinical implications and application

These findings provide a practical framework for incorporating both traditional and emerging risk factors into the clinical management of IAs. In particular, our data highlight the potential role of inflammatory and coagulation biomarkers in daily practice, such as D-dimer, fibrinogen, and LDH. Elevated D-dimer and fibrinogen may signify a hypercoagulable state, suggesting heightened aneurysm wall instability, while elevated LDH reflects cellular damage and metabolic stress. Patients with notably high biomarker levels could warrant more frequent imaging surveillance or earlier intervention, particularly when other risk factors (e.g., younger age, female sex) are present.

We also developed a nomogram that integrates demographic variables, biomarker levels, and other clinical parameters to estimate individual rupture risk. This tool can help clinicians balance the benefits of early intervention against the risks of continued observation. For instance, a patient with moderately elevated D-dimer, younger age, and female sex would likely receive a higher risk score, prompting more vigilant monitoring or expedited treatment.

Additionally, our results underscore the importance of optimizing modifiable factors such as blood pressure control, electrolyte balance (especially potassium), and broader metabolic health. Addressing these factors can further reduce rupture risk and improve outcomes in patients with unruptured IAs. While prospective studies are needed to validate biomarker thresholds and refine the nomogram, our findings lay a foundation for more individualized and proactive IA management.

### 4.6 Limitations

This study has several limitations. First, it was conducted at a single center in China with a predominantly Asian population, which may limit the generalizability of our findings to other ethnic groups and regions. Second, the retrospective design inevitably introduces biases, particularly selection bias and recall bias. We specifically excluded patients with incomplete medical records or those managed conservatively, skewing our sample toward higher-risk cases. Reliance on previously documented data may also lead to inaccuracies in recording lifestyle factors such as smoking or alcohol use. Third, we were unable to obtain precise aneurysm-specific measurements (e.g., exact size, detailed morphology) because comprehensive imaging data were not consistently available, restricting our ability to fully account for these established determinants of rupture risk. Finally, although we employed strict, uniform inclusion criteria and extracted all data from a centralized electronic system to reduce heterogeneity, these measures do not overcome the inherent constraints of a retrospective analysis. Prospective, multicenter studies with standardized data collection, more diverse patient cohorts, and serial imaging assessments are warranted to validate our findings and further elucidate how clinical risk factors interact to influence intracranial aneurysm rupture.

## 5 Conclusion

In conclusion, this study underscores the complex interplay of demographic, inflammatory, metabolic, and coagulation factors in aneurysm rupture risk. The unique findings regarding potassium, creatinine, hematocrit, and LDH, although not widely studied in aneurysm literature, offer potential insights based on mechanisms established in other vascular contexts. These insights may guide future research and support comprehensive risk assessment strategies in aneurysm management.

## Data Availability

The original contributions presented in the study are included in the article/Supplementary material, further inquiries can be directed to the corresponding author.
