# Supplementary material for: Comprehensive analysis of risk factors for intracranial aneurysm rupture: a retrospective cohort study
Source: Front Neurol. 2025 Mar 31;16:1559484. doi: 10.3389/fneur.2025.1559484 (PMC11994311; doi:10.3389/fneur.2025.1559484)
Supplement: Supplementary file 1 [file Presentation_1.pptx]

## Slide 1
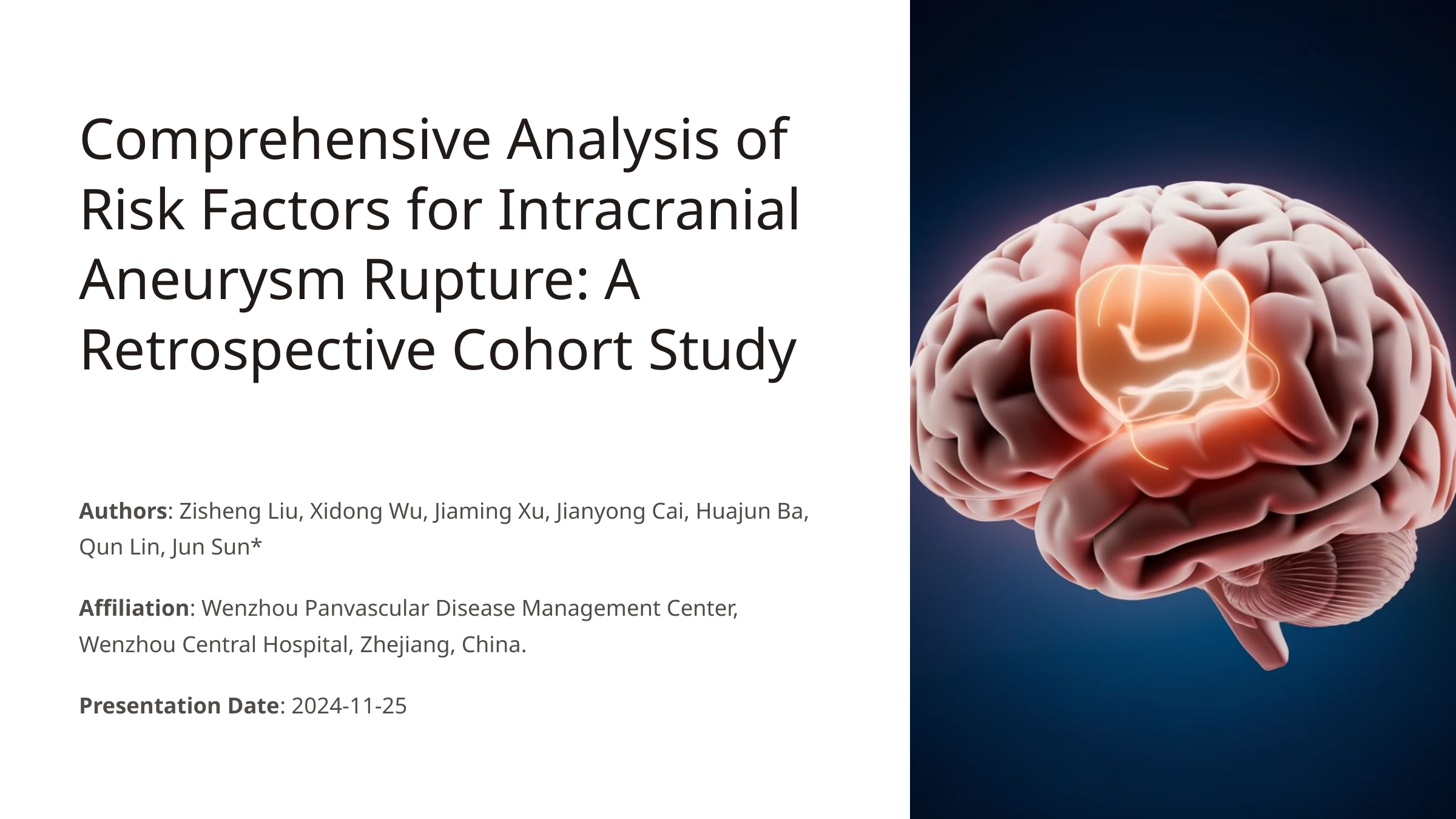

Comprehensive Analysis of Risk Factors for Intracranial Aneurysm Rupture: A Retrospective Cohort Study
Authors: Zisheng Liu, Xidong Wu, Jiaming Xu, Jianyong Cai, Huajun Ba, Qun Lin, Jun Sun*
Affiliation: Wenzhou Panvascular Disease Management Center, Wenzhou Central Hospital, Zhejiang, China.
Presentation Date: 2024-11-25

## Slide 2
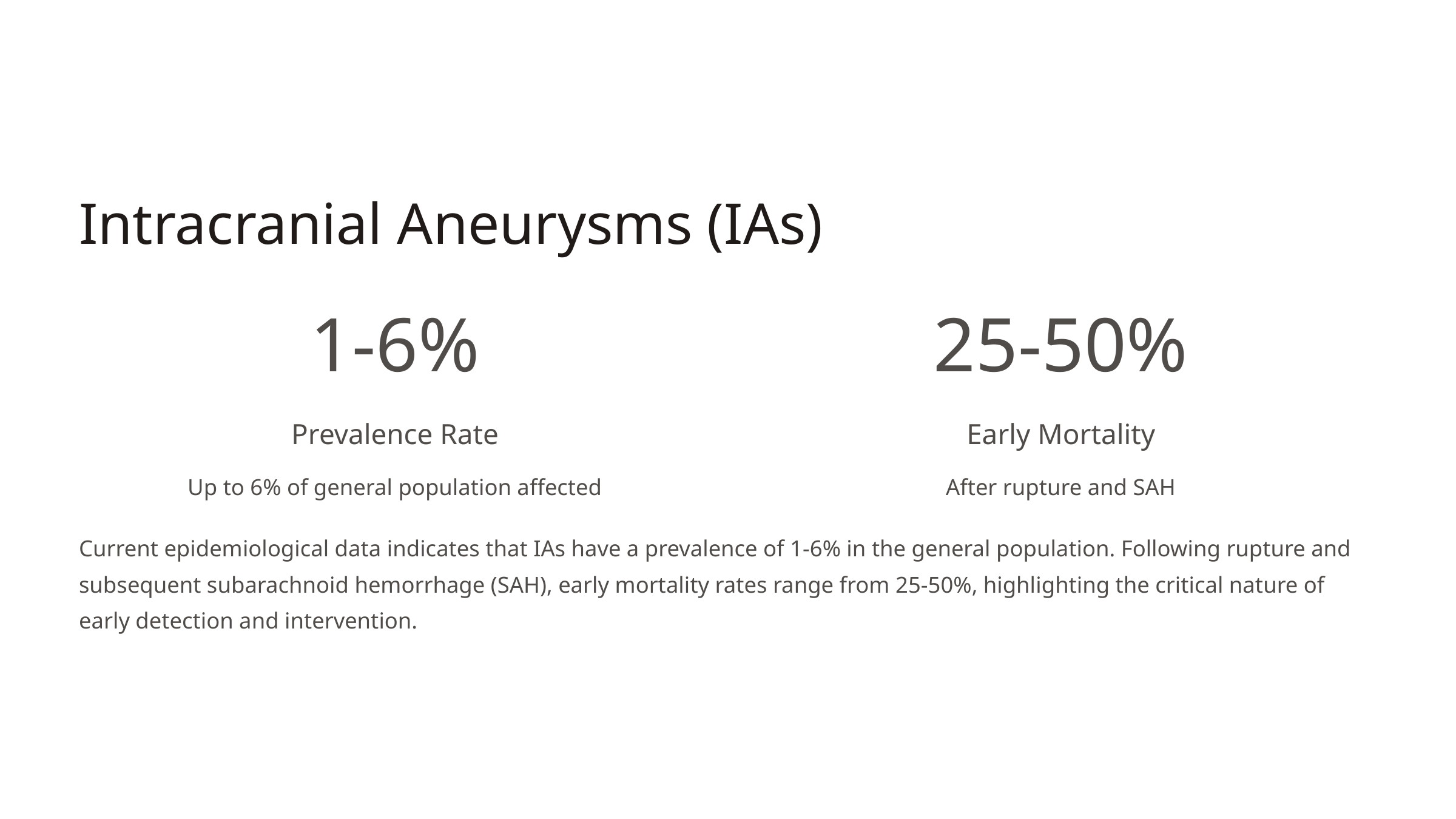

Intracranial Aneurysms (IAs)
1-6%
25-50%
Prevalence Rate
Early Mortality
Up to 6% of general population affected
After rupture and SAH
Current epidemiological data indicates that IAs have a prevalence of 1-6% in the general population. Following rupture and subsequent subarachnoid hemorrhage (SAH), early mortality rates range from 25-50%, highlighting the critical nature of early detection and intervention.

## Slide 3
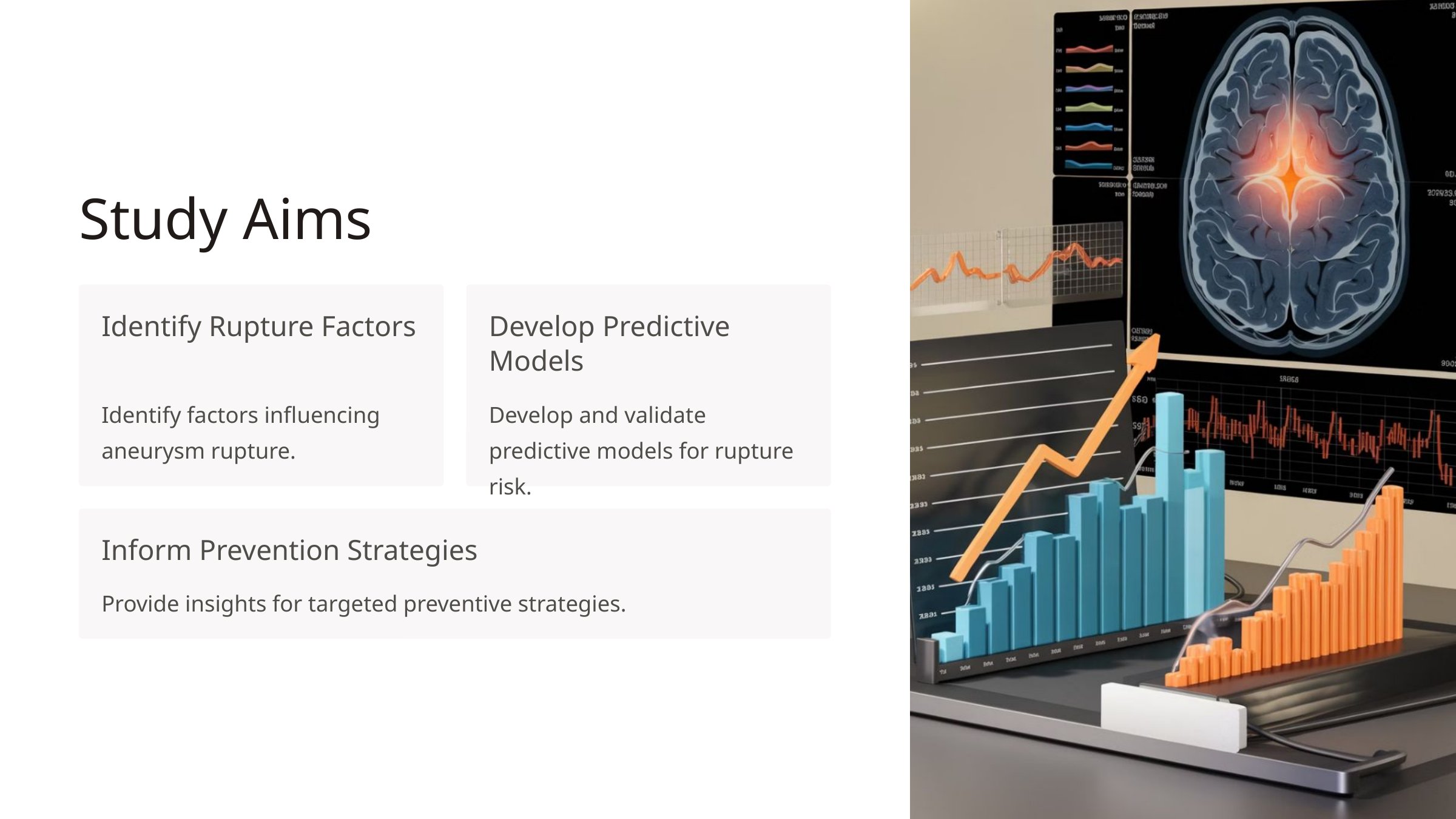

Study Aims
Identify Rupture Factors
Develop Predictive Models
Identify factors influencing aneurysm rupture.
Develop and validate predictive models for rupture risk.
Inform Prevention Strategies
Provide insights for targeted preventive strategies.

## Slide 4
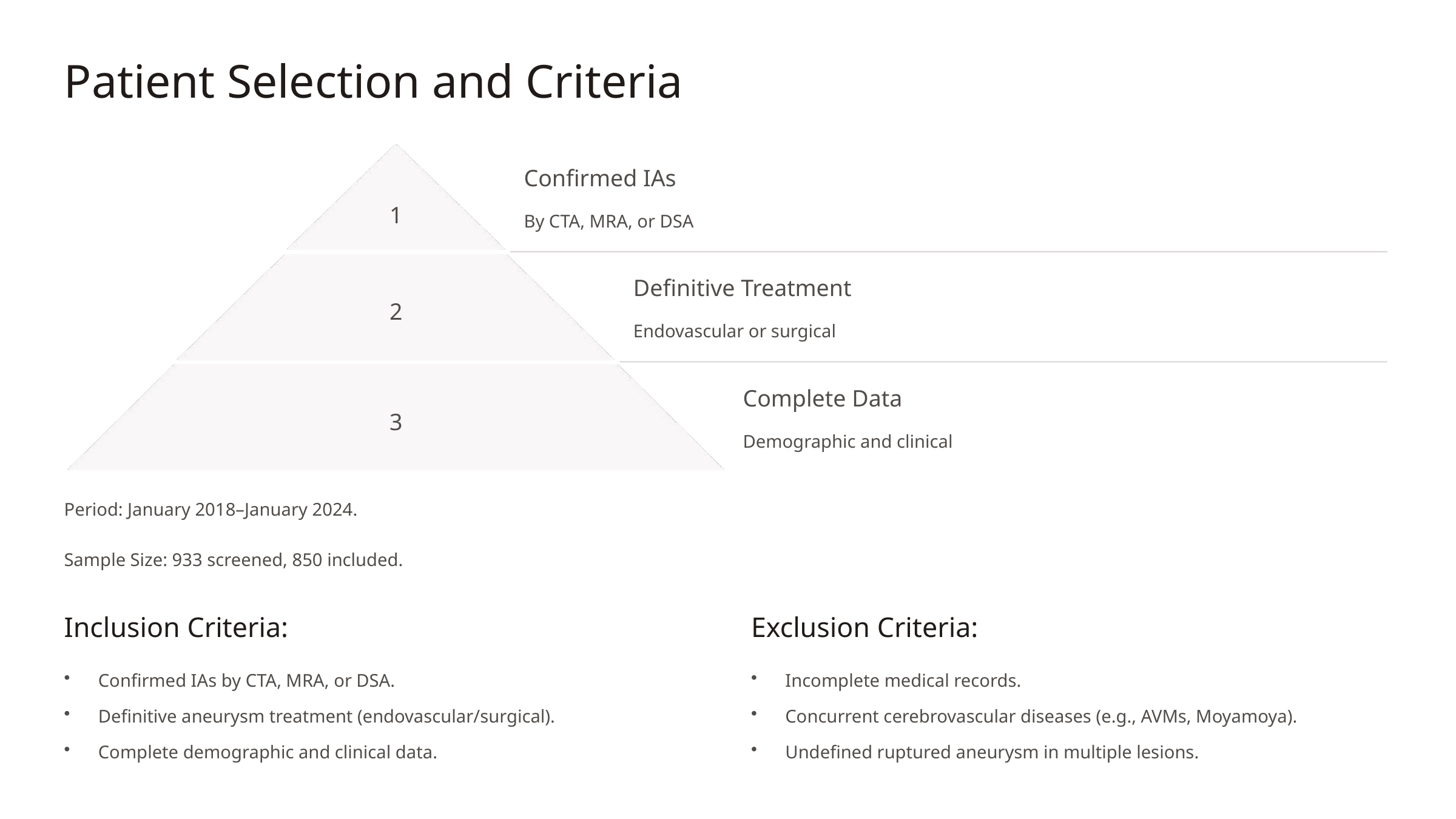

Patient Selection and Criteria
Confirmed IAs
1
By CTA, MRA, or DSA
Definitive Treatment
2
Endovascular or surgical
Complete Data
3
Demographic and clinical
Period: January 2018–January 2024.
Sample Size: 933 screened, 850 included.
Inclusion Criteria:
Exclusion Criteria:
Confirmed IAs by CTA, MRA, or DSA.
Incomplete medical records.
Definitive aneurysm treatment (endovascular/surgical).
Concurrent cerebrovascular diseases (e.g., AVMs, Moyamoya).
Complete demographic and clinical data.
Undefined ruptured aneurysm in multiple lesions.

## Slide 5
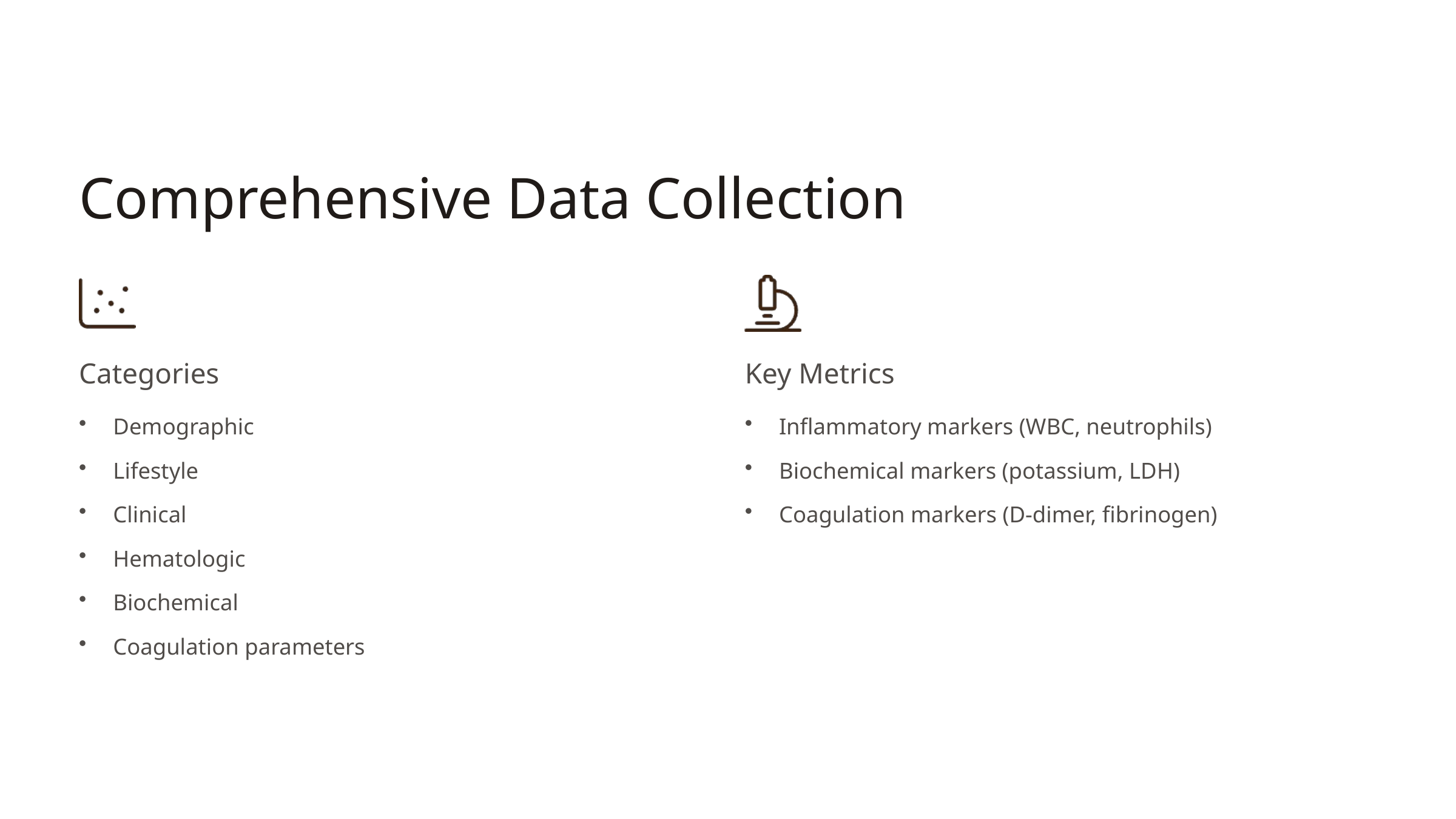

Comprehensive Data Collection
Categories
Key Metrics
Demographic
Inflammatory markers (WBC, neutrophils)
Lifestyle
Biochemical markers (potassium, LDH)
Clinical
Coagulation markers (D-dimer, fibrinogen)
Hematologic
Biochemical
Coagulation parameters

## Slide 6
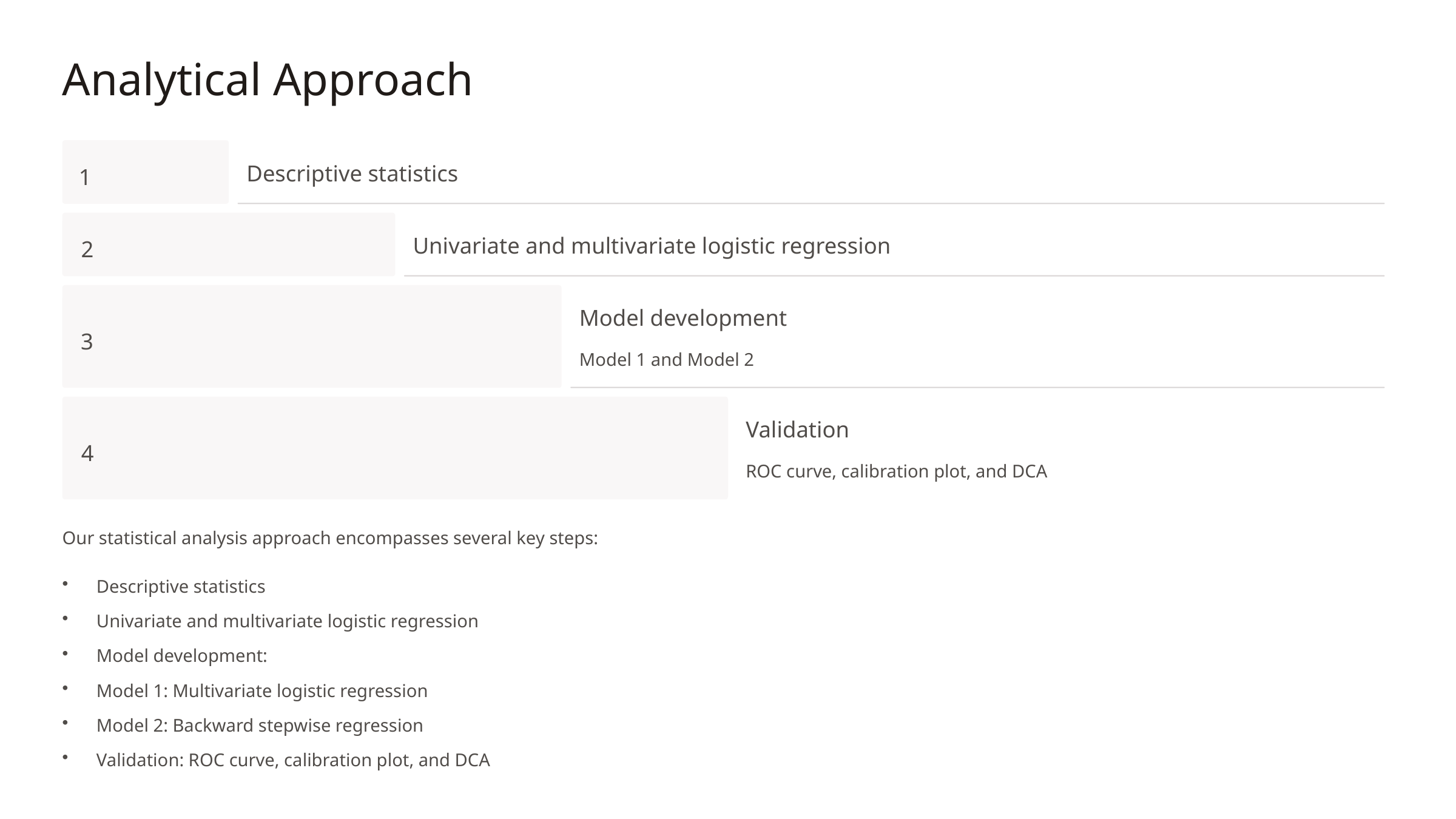

Analytical Approach
1
Descriptive statistics
2
Univariate and multivariate logistic regression
Model development
3
Model 1 and Model 2
Validation
4
ROC curve, calibration plot, and DCA
Our statistical analysis approach encompasses several key steps:
Descriptive statistics
Univariate and multivariate logistic regression
Model development:
Model 1: Multivariate logistic regression
Model 2: Backward stepwise regression
Validation: ROC curve, calibration plot, and DCA

## Slide 7
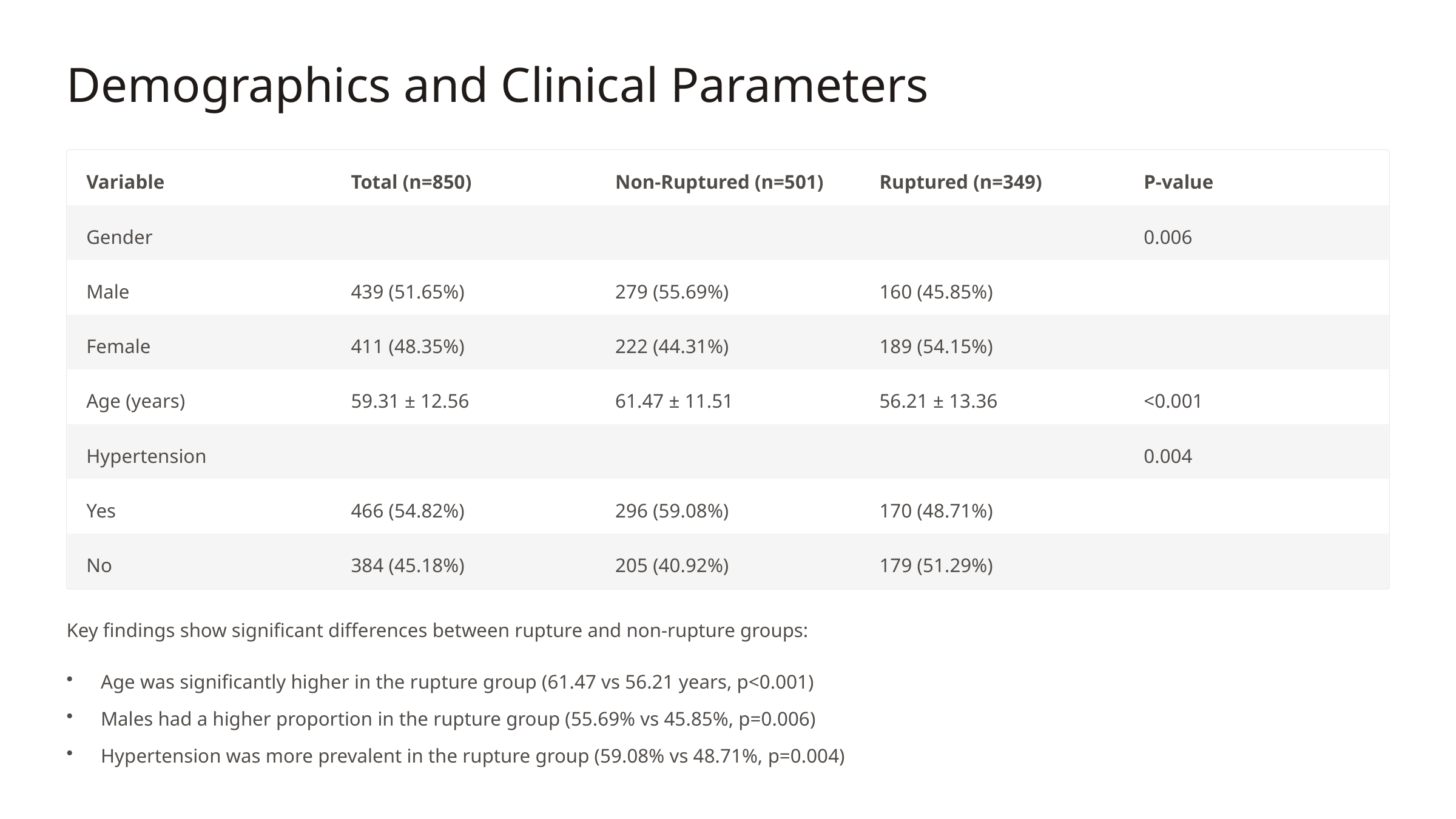

Demographics and Clinical Parameters
Variable
Total (n=850)
Non-Ruptured (n=501)
Ruptured (n=349)
P-value
Gender
0.006
Male
439 (51.65%)
279 (55.69%)
160 (45.85%)
Female
411 (48.35%)
222 (44.31%)
189 (54.15%)
Age (years)
59.31 ± 12.56
61.47 ± 11.51
56.21 ± 13.36
<0.001
Hypertension
0.004
Yes
466 (54.82%)
296 (59.08%)
170 (48.71%)
No
384 (45.18%)
205 (40.92%)
179 (51.29%)
Key findings show significant differences between rupture and non-rupture groups:
Age was significantly higher in the rupture group (61.47 vs 56.21 years, p<0.001)
Males had a higher proportion in the rupture group (55.69% vs 45.85%, p=0.006)
Hypertension was more prevalent in the rupture group (59.08% vs 48.71%, p=0.004)

## Slide 8
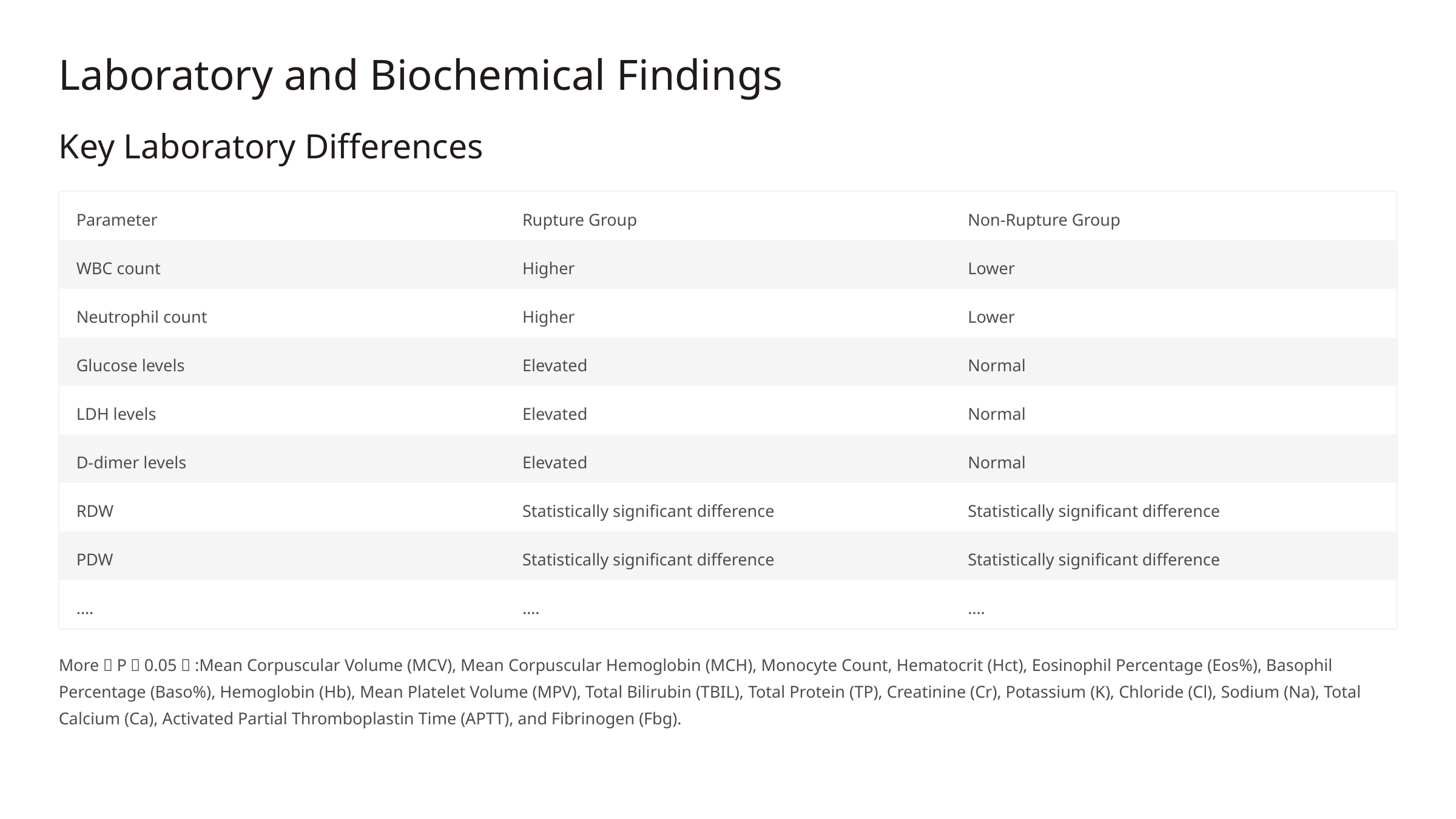

Laboratory and Biochemical Findings
Key Laboratory Differences
Parameter
Rupture Group
Non-Rupture Group
WBC count
Higher
Lower
Neutrophil count
Higher
Lower
Glucose levels
Elevated
Normal
LDH levels
Elevated
Normal
D-dimer levels
Elevated
Normal
RDW
Statistically significant difference
Statistically significant difference
PDW
Statistically significant difference
Statistically significant difference
….
….
….
More（P＜0.05）:Mean Corpuscular Volume (MCV), Mean Corpuscular Hemoglobin (MCH), Monocyte Count, Hematocrit (Hct), Eosinophil Percentage (Eos%), Basophil Percentage (Baso%), Hemoglobin (Hb), Mean Platelet Volume (MPV), Total Bilirubin (TBIL), Total Protein (TP), Creatinine (Cr), Potassium (K), Chloride (Cl), Sodium (Na), Total Calcium (Ca), Activated Partial Thromboplastin Time (APTT), and Fibrinogen (Fbg).

## Slide 9
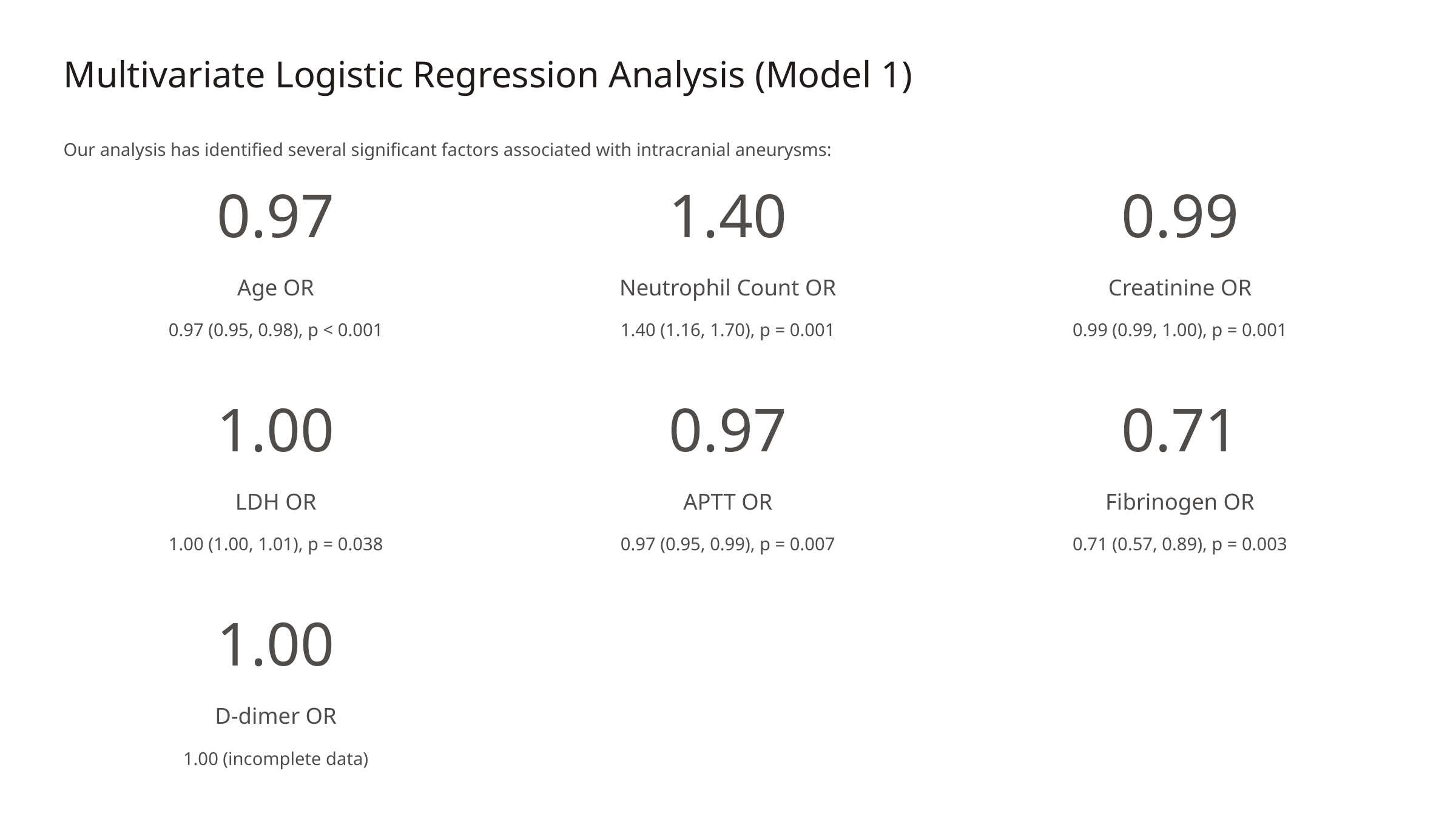

Multivariate Logistic Regression Analysis (Model 1)
Our analysis has identified several significant factors associated with intracranial aneurysms:
0.97
1.40
0.99
Age OR
Neutrophil Count OR
Creatinine OR
0.97 (0.95, 0.98), p < 0.001
1.40 (1.16, 1.70), p = 0.001
0.99 (0.99, 1.00), p = 0.001
1.00
0.97
0.71
LDH OR
APTT OR
Fibrinogen OR
1.00 (1.00, 1.01), p = 0.038
0.97 (0.95, 0.99), p = 0.007
0.71 (0.57, 0.89), p = 0.003
1.00
D-dimer OR
1.00 (incomplete data)

## Slide 10
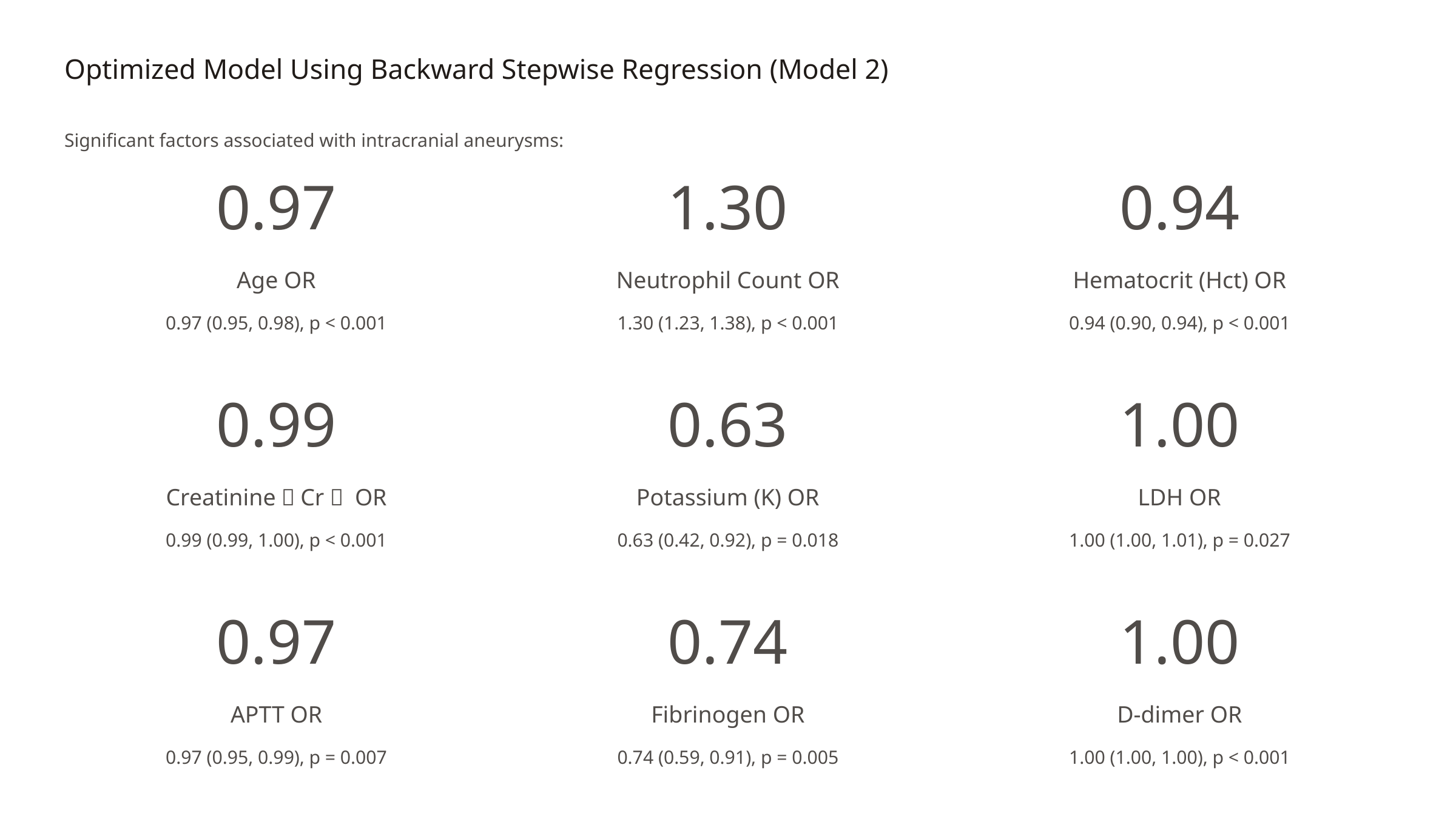

Optimized Model Using Backward Stepwise Regression (Model 2)
Significant factors associated with intracranial aneurysms:
0.97
1.30
0.94
Age OR
Neutrophil Count OR
Hematocrit (Hct) OR
0.97 (0.95, 0.98), p < 0.001
1.30 (1.23, 1.38), p < 0.001
0.94 (0.90, 0.94), p < 0.001
0.99
0.63
1.00
Creatinine（Cr） OR
Potassium (K) OR
LDH OR
0.99 (0.99, 1.00), p < 0.001
0.63 (0.42, 0.92), p = 0.018
1.00 (1.00, 1.01), p = 0.027
0.97
0.74
1.00
APTT OR
Fibrinogen OR
D-dimer OR
0.97 (0.95, 0.99), p = 0.007
0.74 (0.59, 0.91), p = 0.005
1.00 (1.00, 1.00), p < 0.001

## Slide 11
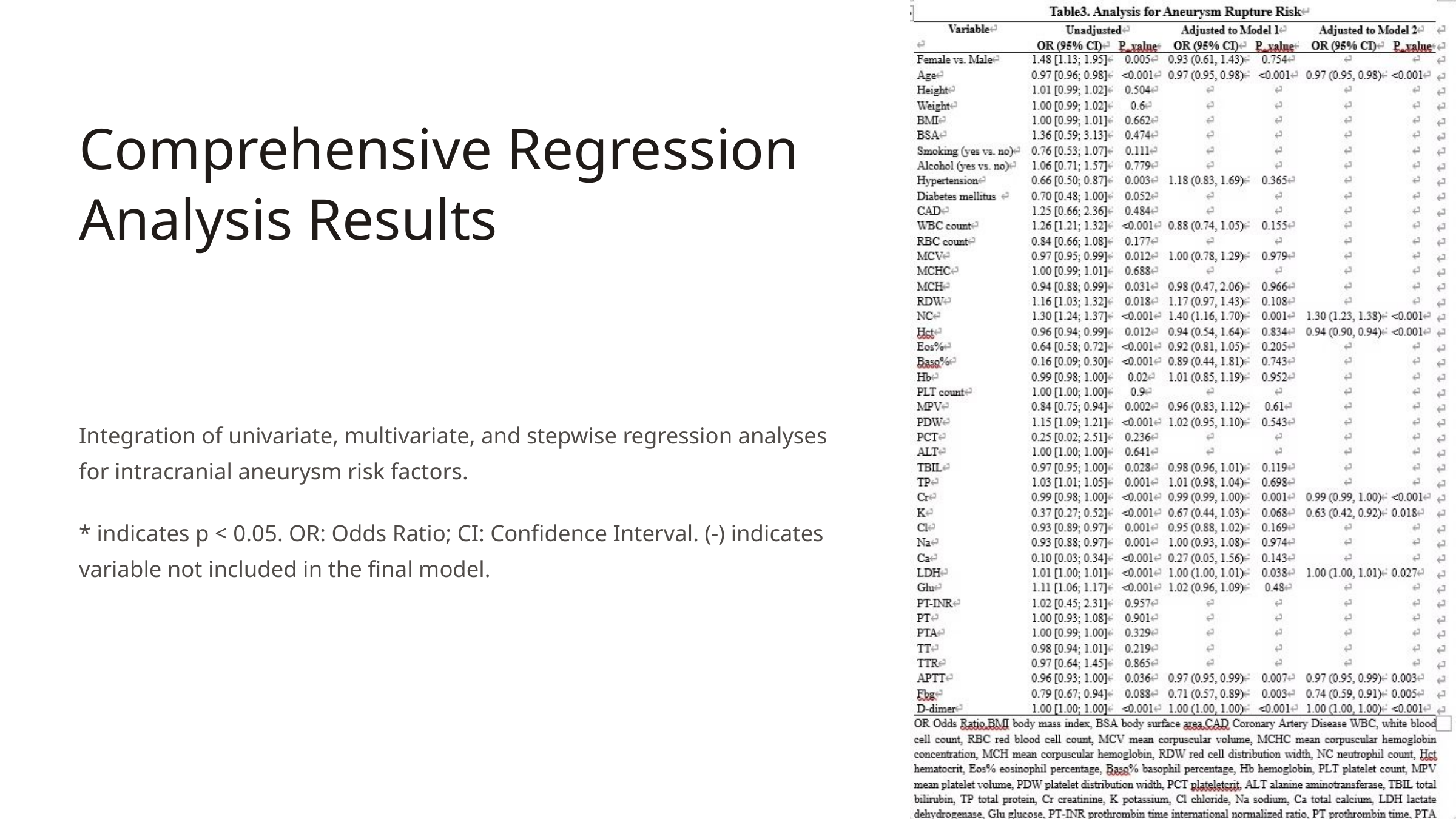

Comprehensive Regression Analysis Results
Integration of univariate, multivariate, and stepwise regression analyses for intracranial aneurysm risk factors.
* indicates p < 0.05. OR: Odds Ratio; CI: Confidence Interval. (-) indicates variable not included in the final model.

## Slide 12
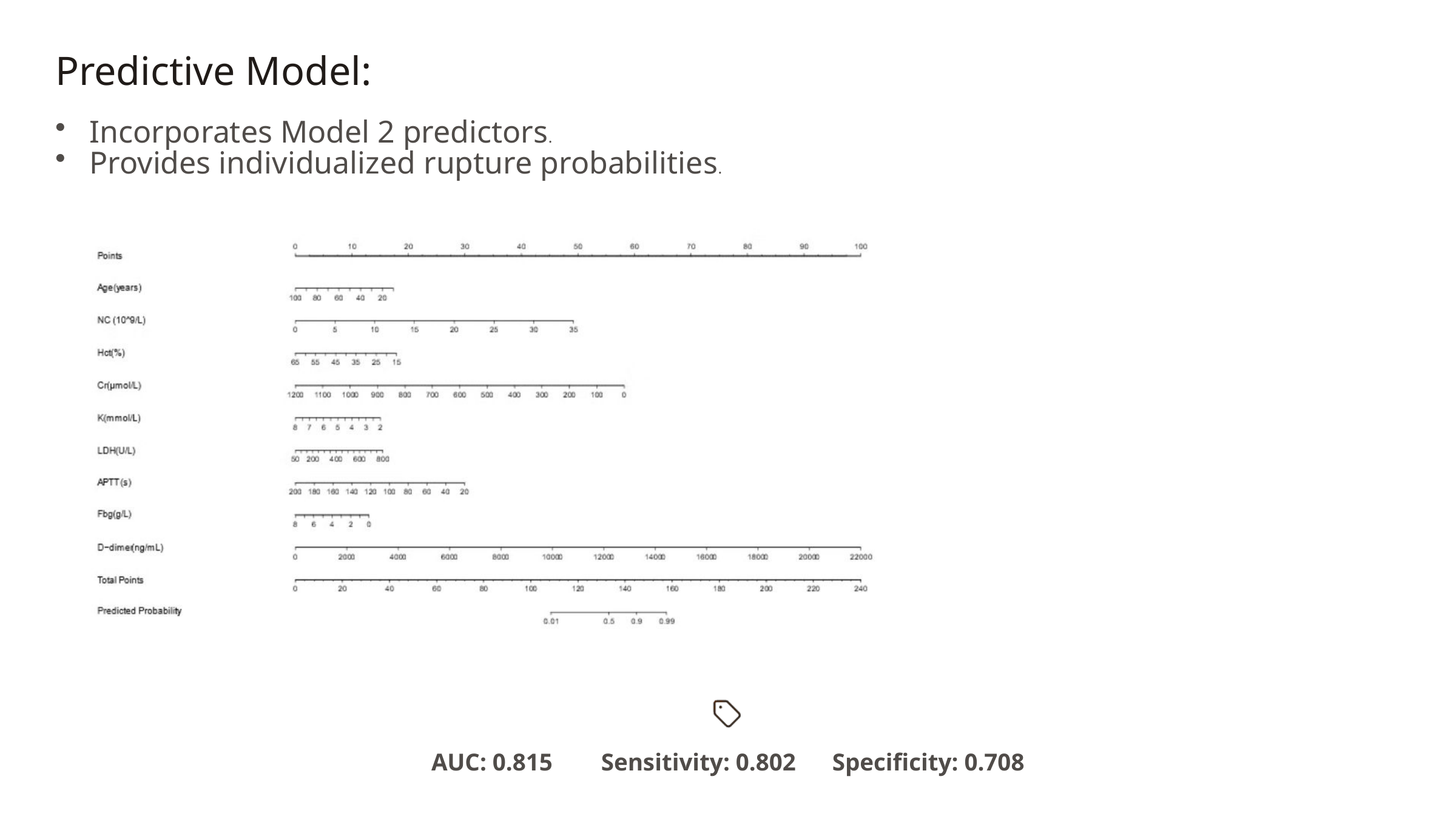

Predictive Model:
Incorporates Model 2 predictors.
Provides individualized rupture probabilities.
AUC: 0.815 Sensitivity: 0.802 Specificity: 0.708

## Slide 13
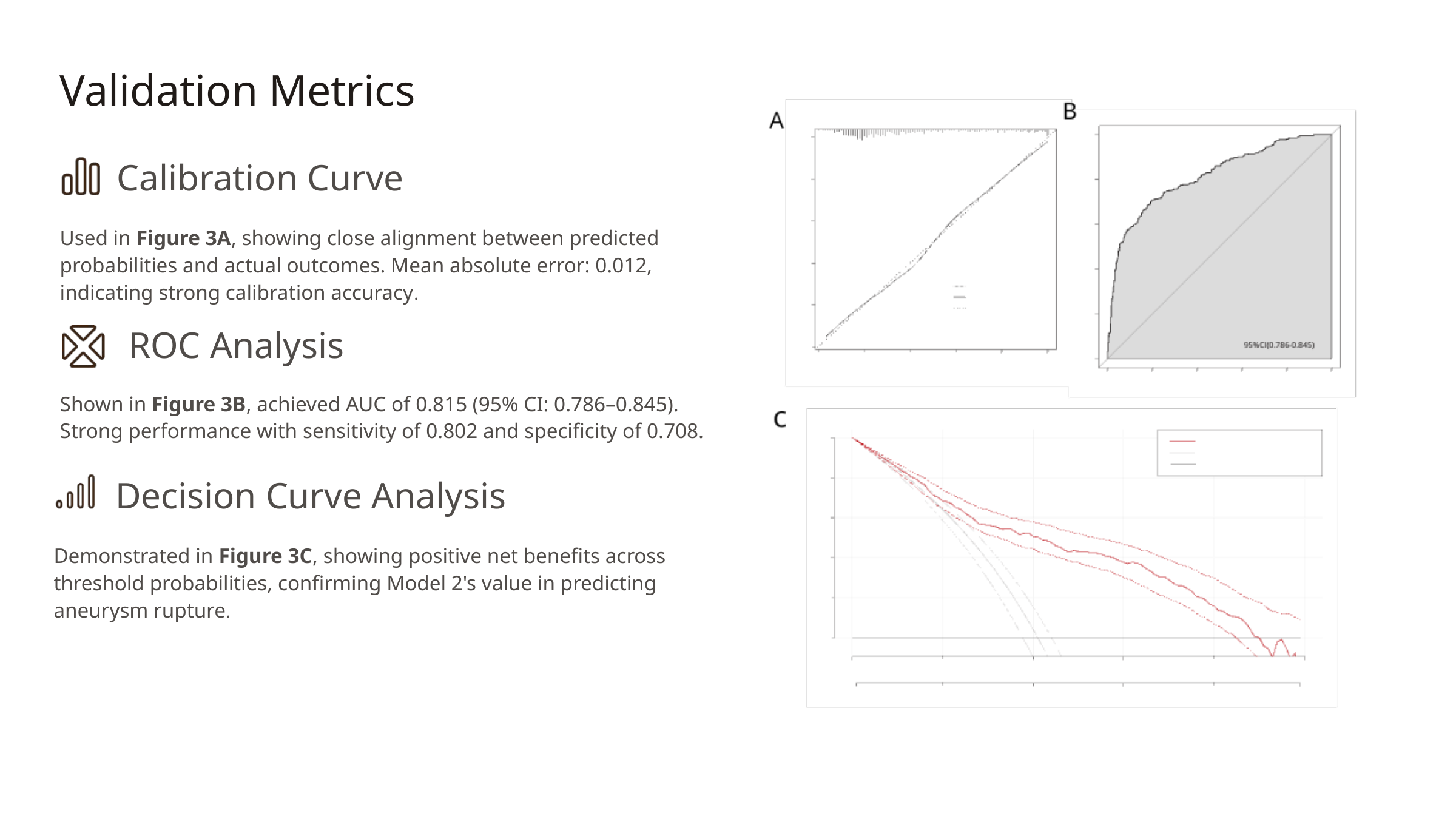

Validation Metrics
Calibration Curve
Used in Figure 3A, showing close alignment between predicted probabilities and actual outcomes. Mean absolute error: 0.012, indicating strong calibration accuracy.
ROC Analysis
Shown in Figure 3B, achieved AUC of 0.815 (95% CI: 0.786–0.845). Strong performance with sensitivity of 0.802 and specificity of 0.708.
Decision Curve Analysis
Demonstrated in Figure 3C, showing positive net benefits across threshold probabilities, confirming Model 2's value in predicting aneurysm rupture.

## Slide 14
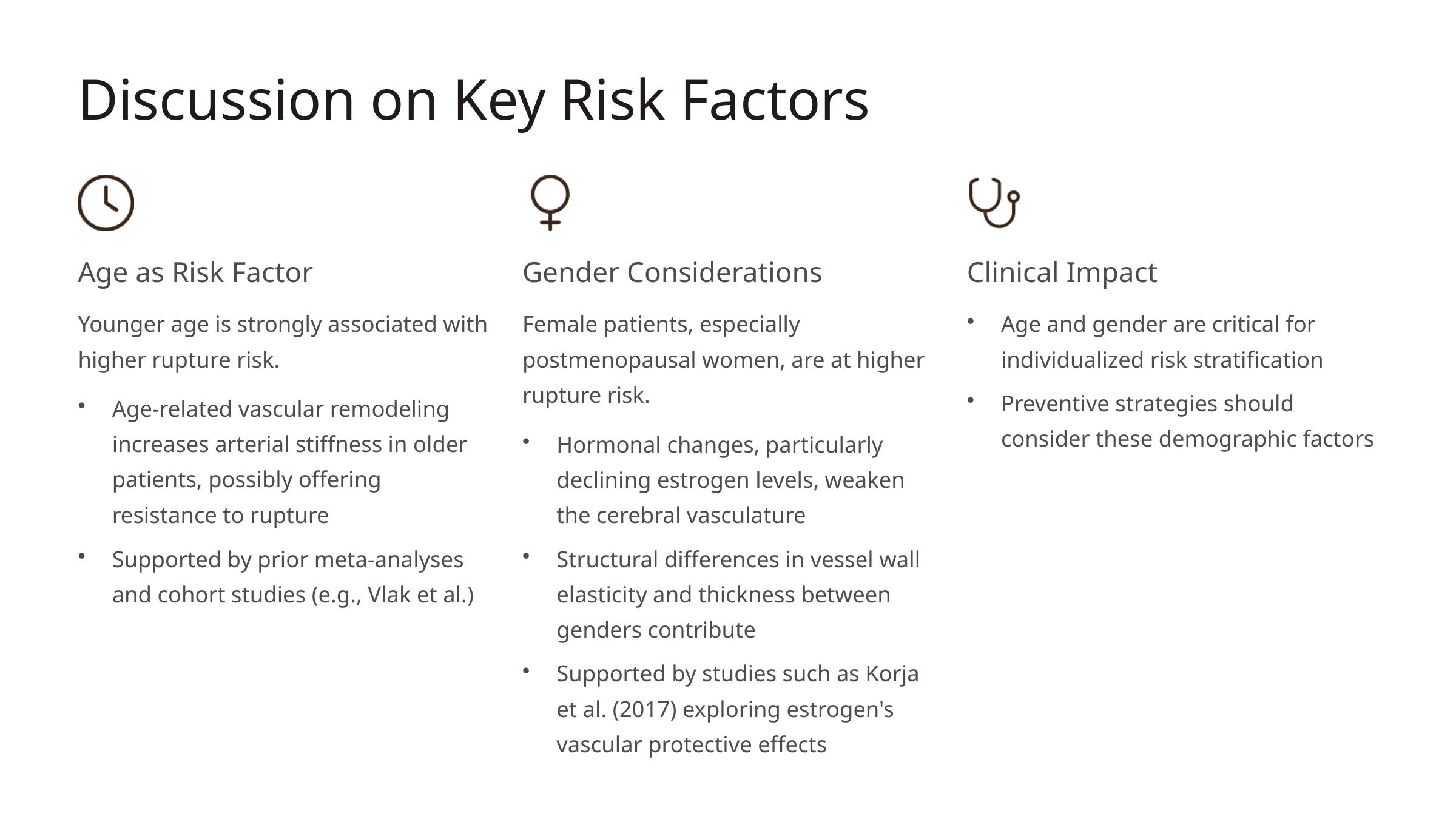

Discussion on Key Risk Factors
Age as Risk Factor
Gender Considerations
Clinical Impact
Younger age is strongly associated with higher rupture risk.
Female patients, especially postmenopausal women, are at higher rupture risk.
Age and gender are critical for individualized risk stratification
Preventive strategies should consider these demographic factors
Age-related vascular remodeling increases arterial stiffness in older patients, possibly offering resistance to rupture
Hormonal changes, particularly declining estrogen levels, weaken the cerebral vasculature
Supported by prior meta-analyses and cohort studies (e.g., Vlak et al.)
Structural differences in vessel wall elasticity and thickness between genders contribute
Supported by studies such as Korja et al. (2017) exploring estrogen's vascular protective effects

## Slide 15
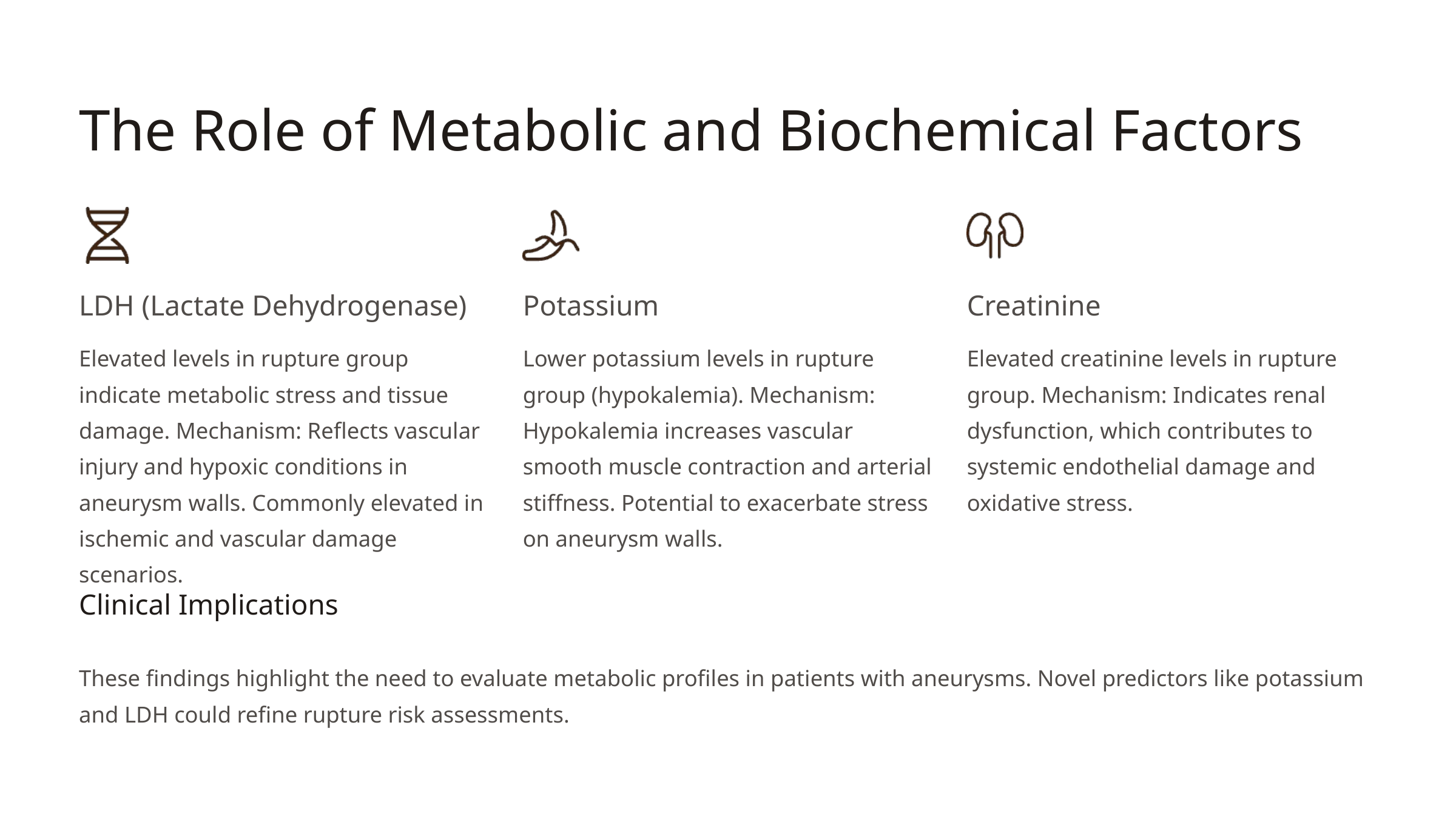

The Role of Metabolic and Biochemical Factors
LDH (Lactate Dehydrogenase)
Potassium
Creatinine
Elevated levels in rupture group indicate metabolic stress and tissue damage. Mechanism: Reflects vascular injury and hypoxic conditions in aneurysm walls. Commonly elevated in ischemic and vascular damage scenarios.
Lower potassium levels in rupture group (hypokalemia). Mechanism: Hypokalemia increases vascular smooth muscle contraction and arterial stiffness. Potential to exacerbate stress on aneurysm walls.
Elevated creatinine levels in rupture group. Mechanism: Indicates renal dysfunction, which contributes to systemic endothelial damage and oxidative stress.
Clinical Implications
These findings highlight the need to evaluate metabolic profiles in patients with aneurysms. Novel predictors like potassium and LDH could refine rupture risk assessments.

## Slide 16
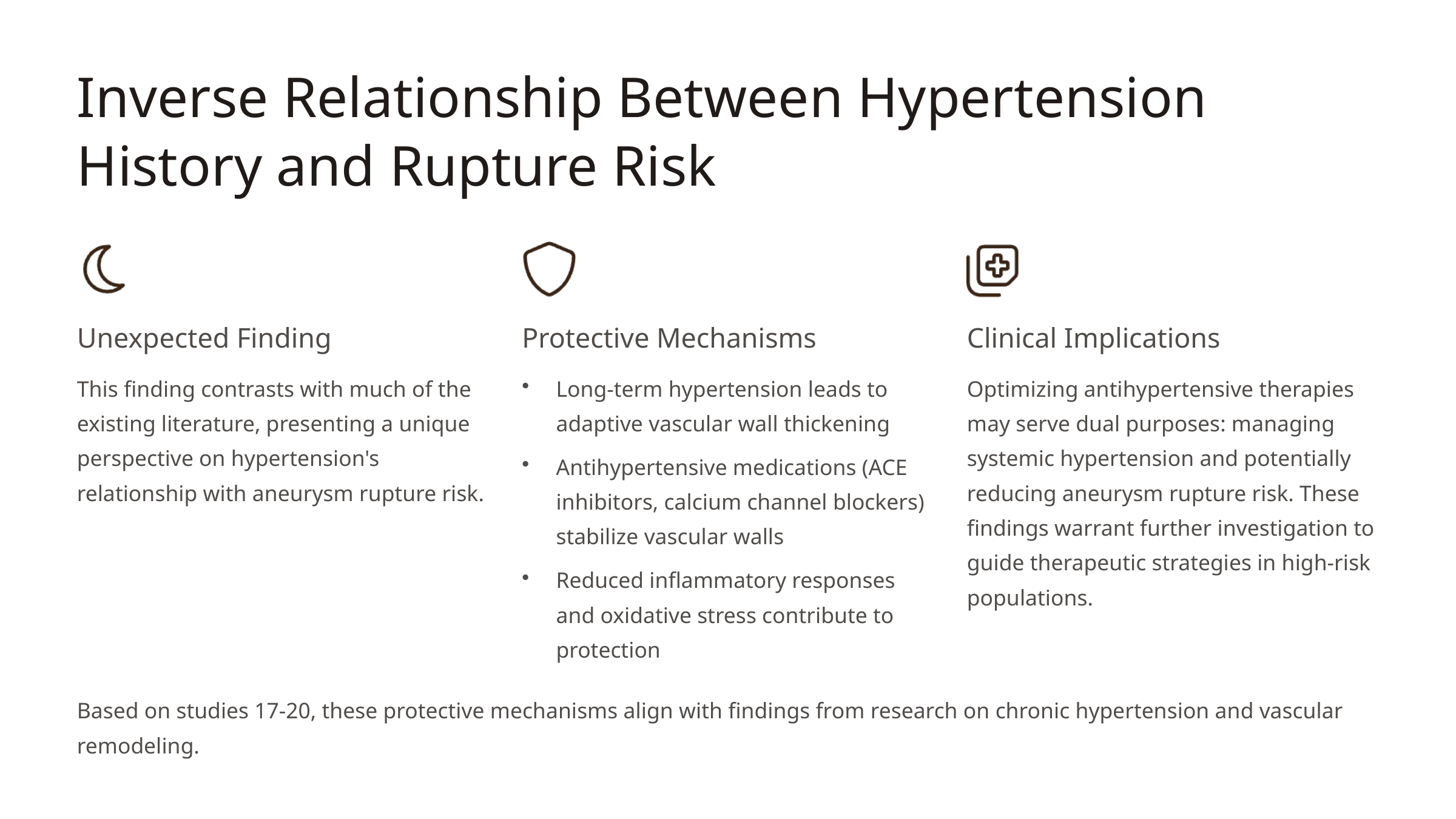

Inverse Relationship Between Hypertension History and Rupture Risk
Unexpected Finding
Protective Mechanisms
Clinical Implications
This finding contrasts with much of the existing literature, presenting a unique perspective on hypertension's relationship with aneurysm rupture risk.
Long-term hypertension leads to adaptive vascular wall thickening
Optimizing antihypertensive therapies may serve dual purposes: managing systemic hypertension and potentially reducing aneurysm rupture risk. These findings warrant further investigation to guide therapeutic strategies in high-risk populations.
Antihypertensive medications (ACE inhibitors, calcium channel blockers) stabilize vascular walls
Reduced inflammatory responses and oxidative stress contribute to protection
Based on studies 17-20, these protective mechanisms align with findings from research on chronic hypertension and vascular remodeling.

## Slide 17
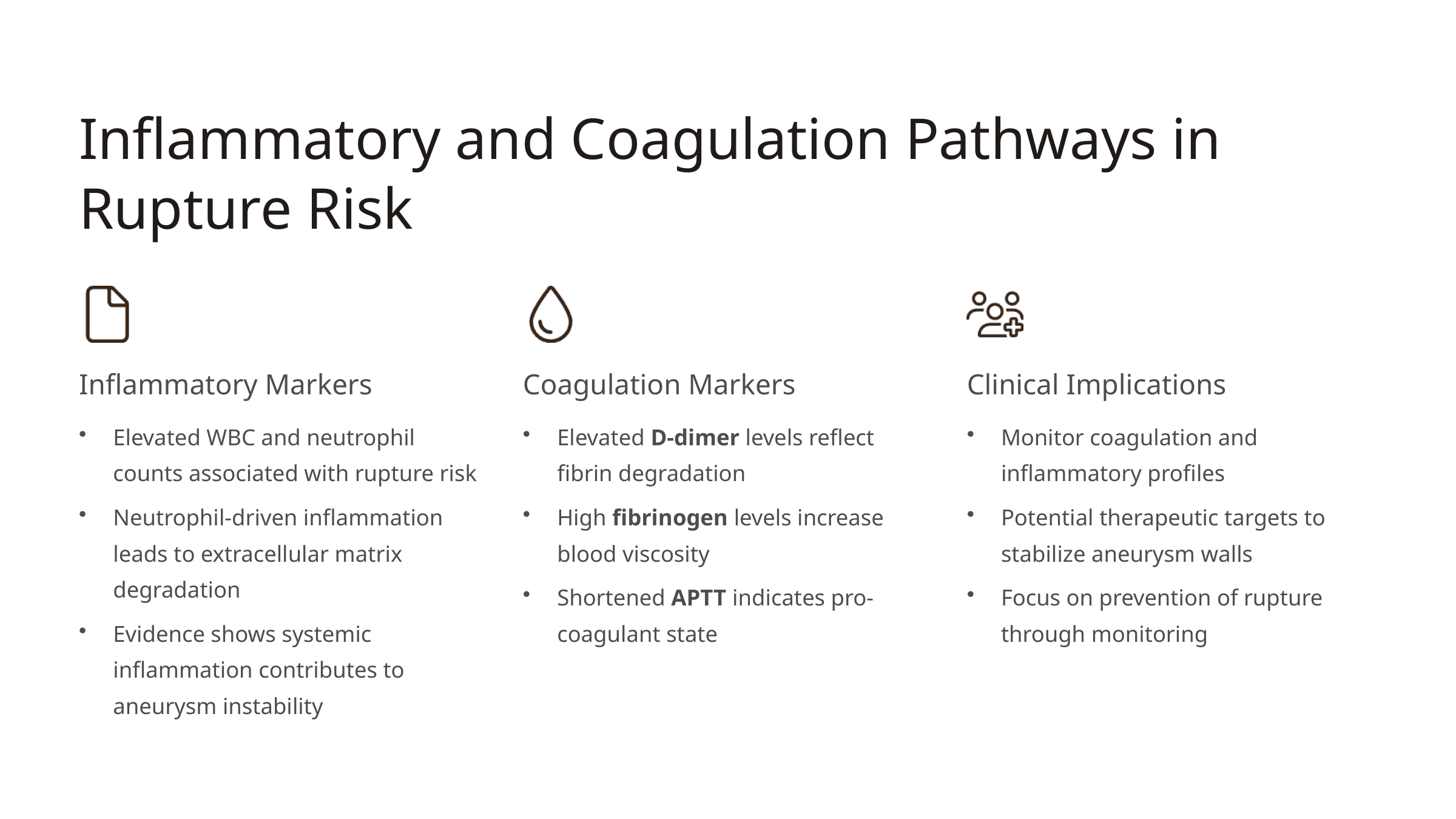

Inflammatory and Coagulation Pathways in Rupture Risk
Inflammatory Markers
Coagulation Markers
Clinical Implications
Elevated WBC and neutrophil counts associated with rupture risk
Elevated D-dimer levels reflect fibrin degradation
Monitor coagulation and inflammatory profiles
Neutrophil-driven inflammation leads to extracellular matrix degradation
High fibrinogen levels increase blood viscosity
Potential therapeutic targets to stabilize aneurysm walls
Shortened APTT indicates pro-coagulant state
Focus on prevention of rupture through monitoring
Evidence shows systemic inflammation contributes to aneurysm instability

## Slide 18
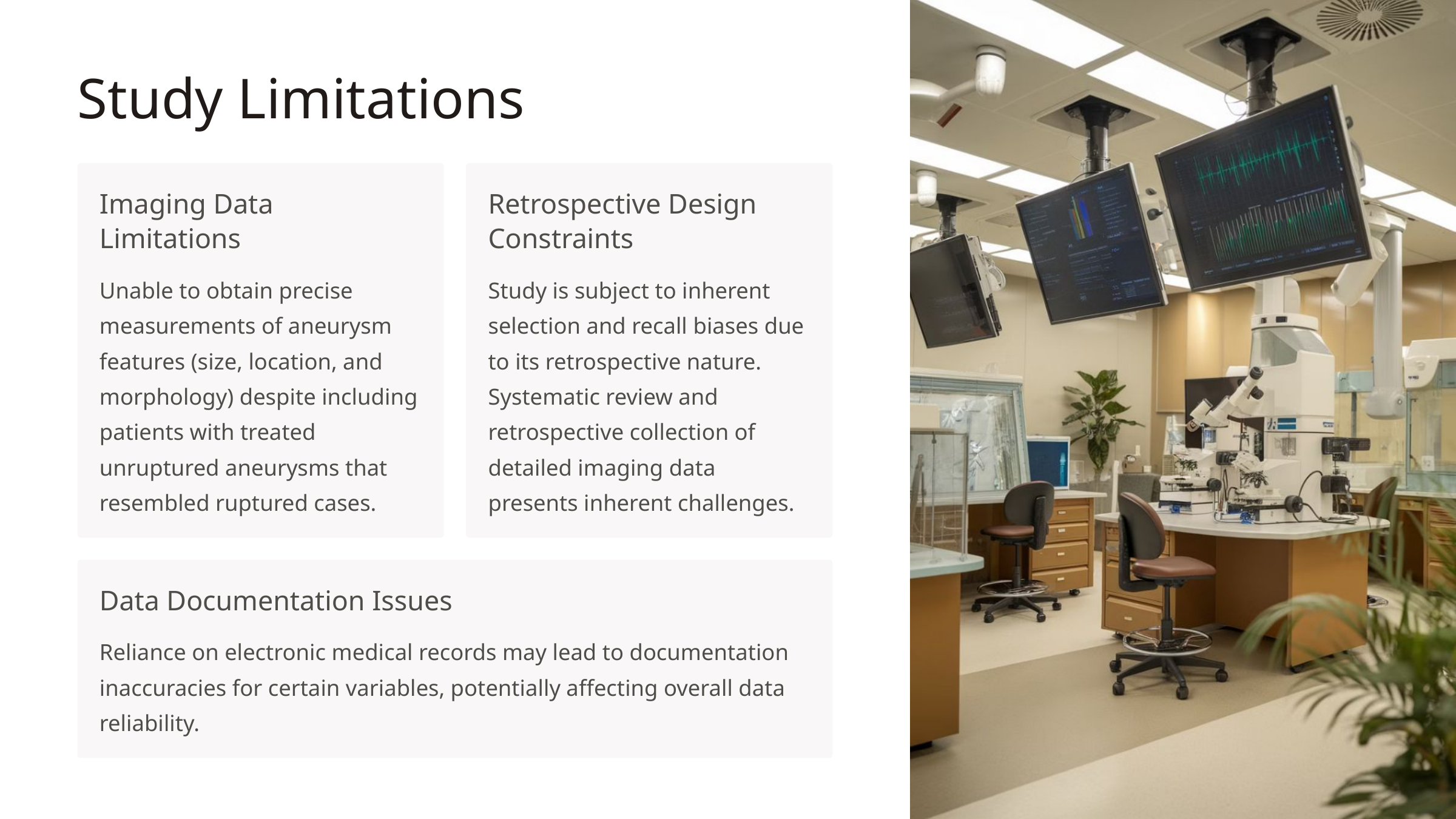

Study Limitations
Imaging Data Limitations
Retrospective Design Constraints
Unable to obtain precise measurements of aneurysm features (size, location, and morphology) despite including patients with treated unruptured aneurysms that resembled ruptured cases.
Study is subject to inherent selection and recall biases due to its retrospective nature. Systematic review and retrospective collection of detailed imaging data presents inherent challenges.
Data Documentation Issues
Reliance on electronic medical records may lead to documentation inaccuracies for certain variables, potentially affecting overall data reliability.

## Slide 19
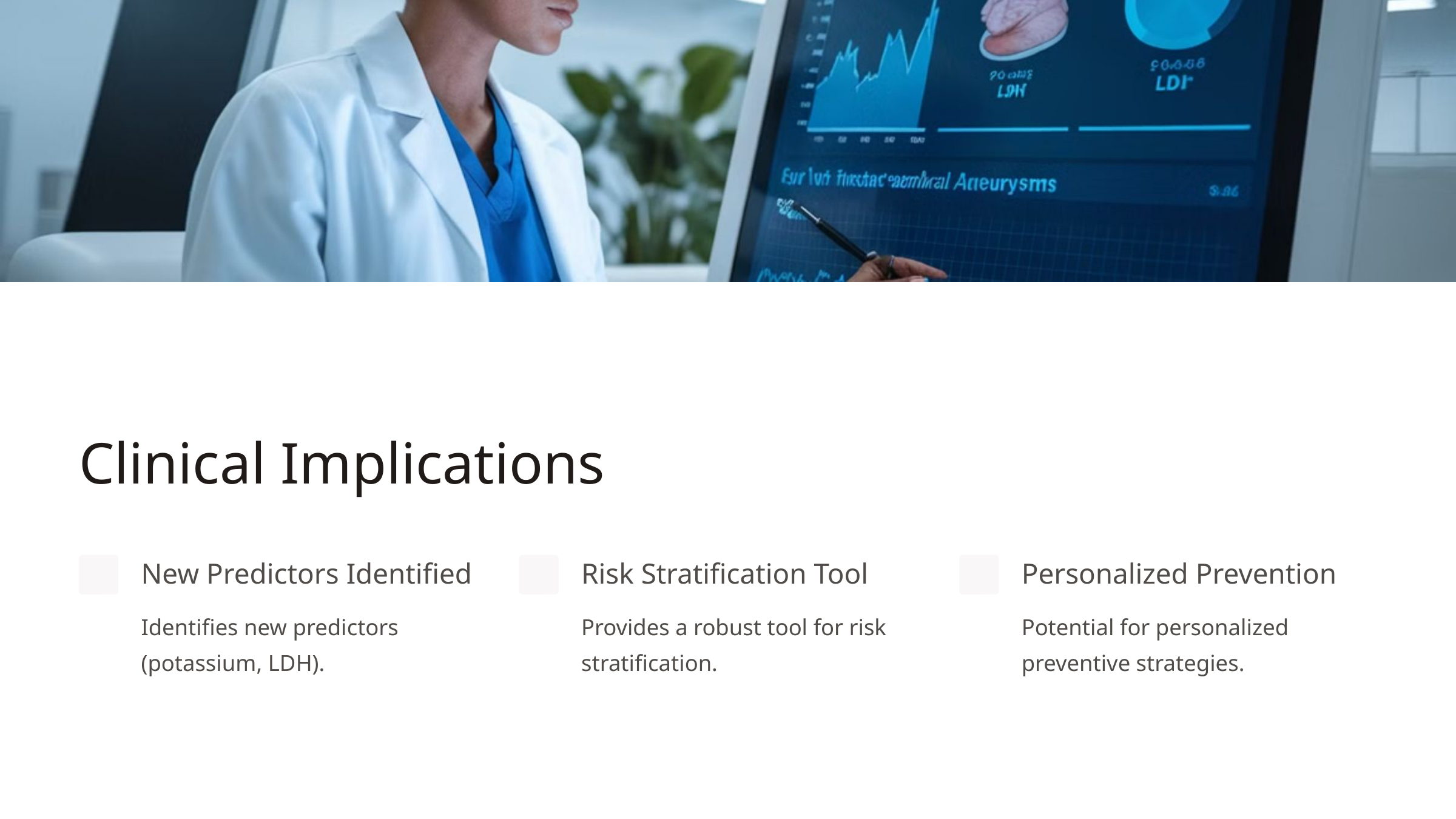

Clinical Implications
New Predictors Identified
Risk Stratification Tool
Personalized Prevention
Identifies new predictors (potassium, LDH).
Provides a robust tool for risk stratification.
Potential for personalized preventive strategies.

## Slide 20
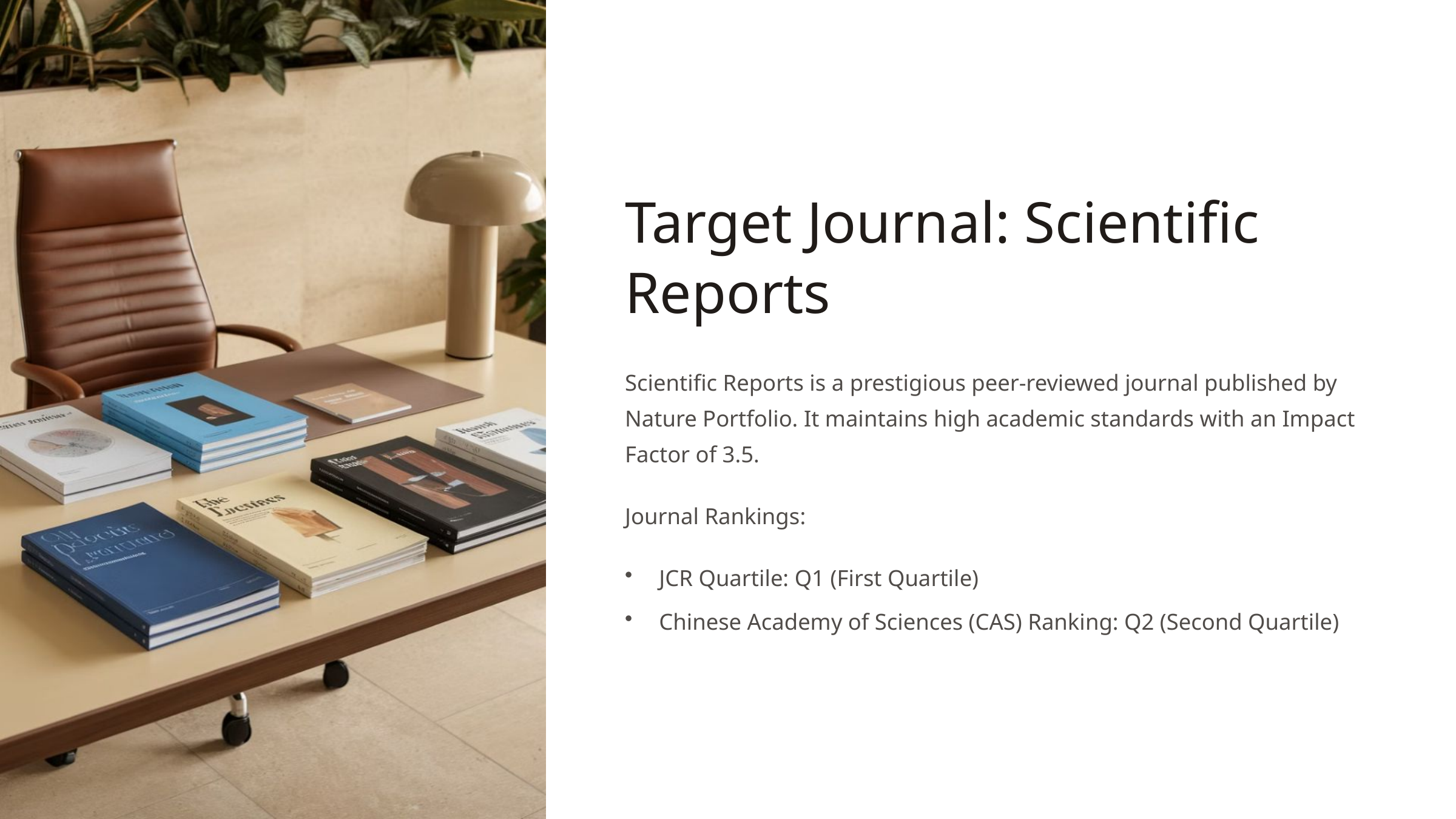

Target Journal: Scientific Reports
Scientific Reports is a prestigious peer-reviewed journal published by Nature Portfolio. It maintains high academic standards with an Impact Factor of 3.5.
Journal Rankings:
JCR Quartile: Q1 (First Quartile)
Chinese Academy of Sciences (CAS) Ranking: Q2 (Second Quartile)

## Slide 21
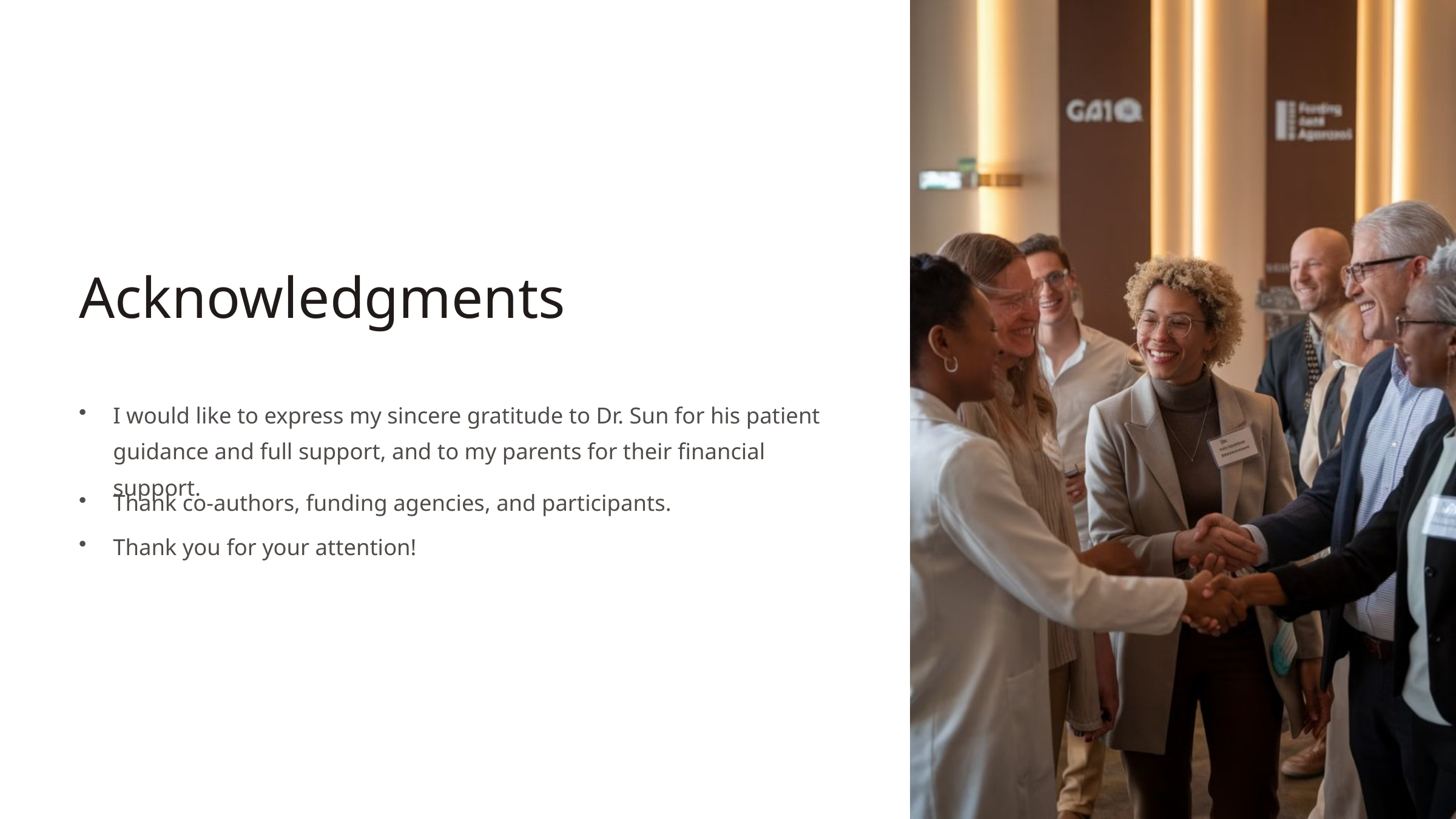

Acknowledgments
I would like to express my sincere gratitude to Dr. Sun for his patient guidance and full support, and to my parents for their financial support.
Thank co-authors, funding agencies, and participants.
Thank you for your attention!
